# Arbuscular mycorrhizal fungi contribute to wheat yield in an agroforestry system with different tree ages

**DOI:** 10.3389/fmicb.2022.1024128

**Published:** 2022-11-15

**Authors:** Xu Qiao, Tao Sun, Junjie Lei, Li Xiao, Lihua Xue, Heng Zhang, Jiyu Jia, Shuikuan Bei

**Affiliations:** ^1^Peking Union Medical College, Institute of Medicinal Plant Development, Chinese Academy of Medical Sciences, Beijing, China; ^2^Key Laboratory of Desert-Oasis Crop Physiology, Ecology and Cultivation, MOARA/Institute of Grain Crops, Xinjiang Academy of Agricultural Sciences, Urumqi, China; ^3^College of Water Conservancy and Civil Engineering, Xinjiang Agricultural University, Urumqi, China; ^4^Institute of Agricultural Resources and Regional Planning, Chinese Academy of Agricultural Sciences, Beijing, China; ^5^Key Laboratory of Plant-Soil Interactions, College of Resources and Environmental Sciences, Ministry of Education, National Academy of Agriculture Green Development, China Agricultural University, Beijing, China

**Keywords:** agroforestry system, soil microbial community, arbuscular mycorrhizal fungi, stand age, yield

## Abstract

Intercropping achieved through agroforestry is increasingly being recognized as a sustainable form of land use. In agroforestry, the roots of trees and crops are intermingled, and their interactions and the production of exudates alter the soil environment and soil microbial community. Although tree–crop interactions vary depending on the stand age of the trees, how stand age affects beneficial microorganisms, including arbuscular mycorrhizal fungi (AMF), and whether changes in soil microorganisms feed back on crop growth in agroforestry systems are unknown. We therefore conducted a long-term field study to compare changes in the soil microbial and AMF communities in a jujube/wheat agroforestry system containing trees of different stand ages: 3-year-old jujube, 8-year-old jujube, and 13-year-old jujube. Our results showed that by changing soil moisture and available phosphorus content, the stand age of the trees had a significant effect on the soil microbial and AMF communities. Soil moisture altered the composition of soil bacteria, in particular the proportions of Gram-positive and Gram-negative species, and available phosphorus had significant effects on the AMF community. A network analysis showed that older stands of trees reduced both AMF diversity and network complexity. An ordinary least squares regression analysis indicated that AMF diversity, network complexity, and stability contributed to wheat yield. Finally, structural equation modeling showed that changes in edaphic factors induced by tree age brought about significant variation in the soil microbial and AMF communities, in turn, affecting crop growth. Our study highlights the crucial roles of soil microorganisms, in particular AMF, in supporting plant growth in agroforestry systems as well as the need to consider stand age in the establishment of these systems.

## Introduction

Agroforestry, in which at least one woody perennial species is grown alongside a crop to increase crop diversity and productivity, is increasingly being recognized as a sustainable form of land use (Fentahun and Hager, 2010; Araujo et al., 2012). The ecological benefits of agroforestry include enhanced carbon sequestration (Baah-Acheamfour et al., 2014), improved soil quality (Dollinger and Jose, 2018), and increased microbial diversity (Bagyaraj et al., 2015; Duchene et al., 2017). Soil microbes regulate multiple ecosystem services, such as nutrient cycling (Rodrigues et al., 2013), organic matter decomposition (Kaiser et al., 2015), plant productivity (Tamburini et al., 2020), and plant disease control (Wei et al., 2019). Nevertheless, the impact of agroforestry on the structure of the soil microbial community is poorly understood, although information on the composition and diversity of microbial community and their determinants is critical to optimizing agroforestry systems (Liu et al., 2019).

The trees in an agroforestry system compete strongly with crops for soil nutrients, sunlight, moisture, and other available resources, which, in turn, could reduce both tree and crop production. The competition for resources increases with increasing tree growth because of the accompanying changes in spatiotemporal light patterns (Tscharntke et al., 2011; Liu et al., 2019). Therefore, in the establishment of an agroforestry system, the stand age of the trees must be taken into account (Peerawat et al., 2018). However, belowground competition would be more important than aboveground competition in many intercropping systems (Remison and Snaydon, 1980). In agroforestry, the roots of trees and crops are intermingled, and their interactions together with the production of exudates could alter both bulk soil and rhizosphere environments, such as the soil organic matter content and pH (Duchene et al., 2017), which would induce significant variation in the soil microbial community. In a study by Liu et al. (2019), bacterial communities in an agroforestry system exhibited obvious horizon-specific seasonal variation in response to spatial and temporal heterogeneity in edaphic factors. Conversely, microbial dynamics feeds back on plant fitness (Philippot et al., 2013; Hutchins et al., 2019). Yet the response of soil microbial communities to tree age, and which microbes in agroforestry systems play a crucial role in affecting plant growth are unknown.

Soil microorganisms, especially of beneficial microbes, modulate a number of processes in agroecosystems. For example, arbuscular mycorrhizal fungi (AMF), which act as key components of the soil microbial community, contribute to the development of healthy soils and agricultural sustainability (Jeffries et al., 2003; Guzman et al., 2021). AMF establishes associations with the majority of terrestrial plants, including most crops (Barrios, 2007; Turrini et al., 2018). In return for receiving carbon and energy from the host plant (Zhu and Miller, 2003; Bryla and Eissenstat, 2005), they provide plants with mineral nutrients (Wipf et al., 2019). Several studies have demonstrated that sustainable management practices, such as intercropping, positively influence the diversity and composition of AMF communities compared to conventional management (Meng et al., 2015). In agroforestry, the high plant diversity achieved with tree-based intercropping promotes the mycorrhizal network (Menezes et al., 2016; van Tuinen et al., 2020). However, little is known about the influence of tree-based intercropping systems on the diversity of AMF communities in tree and crop roots. The consensus is that native trees alter the soil dynamics of their rhizospheres and, in turn, influence the AMF community (Caravaca et al., 2020). And as tree grow, they further change the vegetation cover and the physicochemical attributes, such as soil moisture, available phosphorous, and available potassium, of the soil ecosystem in which mycorrhizas can be active (Sheng et al., 2017; Gao et al., 2019). This implies that the stand age of the trees indirectly shapes the composition of the AMF community and, by changing the properties of the soil, affects the successful establishment of AMF in the crops. In-depth knowledge of the roles of soil microbial and AMF communities in controlling plant productivity in agroforestry systems will help select a stand age that best contributes to the agroforestry system and that is able to maintain ecosystem stability.

Jujube agroforestry is extremely important in China. In the Xinjiang Uygur Autonomous Region (Northwest China), it covers > 1.2 million hectares (Zhang et al., 2014). This area is characterized by with rich light and heat resources, large temperature difference between day and night, which is very suitable for fruit trees. With recent adjustments to planting systems in pursuit of high-yield and high-efficiency crops, jujube trees have been widely planted in farms within Xinjiang Province, China. These intercropping systems have considerable potential to provide food and nutritional security and to contribute to local economic development. However, as a consequence of tree aging, the change of planting structure will inevitably affect the cultivated soil quality and farmland ecological environment in this area, and will further affect the change of soil microbial community structure and function. Therefore, knowledge of the effects of tree age on soil microbial and AMF communities and of the role of these communities in promoting crop growth is critical to ensuring food security in the country. In this study, we conducted a field experiment designed to explore the soil microbial and AMF communities associated with jujube trees of different stand ages in an agroforestry system and resulting functional changes in crop growth. We hypothesized that (1) both the soil microbial community and the AMF community would be significantly affected by tree stand age because of the effects of tree age on soil properties, and (2) that variation in the composition and diversity of the soil microbial and AMF communities would predict changes in crop productivity.

## Materials and methods

### Study sites and sample collection

A field experiment was conducted in 2015 at the 4th Village of Zepu County with an altitude of about 1,300 m (38°05′N, 77°10′E), Kashi Prefecture, Xinjiang Uygur Autonomous Region, China. This area has a typical arid climate with an annual mean temperature of 11.6°C (1961–2008), an annual precipitation of 54.8 mm, and a potential evaporation of 2,079 mm. The mean frost-free period is 212 days.

The three planting patterns consisted of wheat intercropped with 3-year-old (IN3), 8-year-old (IN8), and 13-year-old (IN13) jujube trees (*Ziziphus jujuba Mill. Junzao*) were selected in 2015. Supplementary Figure S1A presents the growth stages of wheat and jujube trees. Jujube trees were planted in a North–South orientation. Basic information for the three jujube tree age groups is shown in Supplementary Table S1. Each intercropping plot included two rows (15 m length) of jujube trees and one wheat strip. There were three replicate plots per planting patterns. In all tree age plots, the row spacing was 500 cm, and there was 150 cm between within a row. The wheat strips in jujube intercropping systems were 3.3 m wide. Minimum distances between trees and wheat rows were 0.85 m for jujube, and intercropped wheat occupied 66% of the total area in the jujube-based system (Supplementary Figure S1B). Date trees are pruned from late February to mid-March in spring, keeping only the main branches and cutting off all the thinner branches. The pruned branches are carried out of the field. Each plot were each 0.4 ha in area and had a density of 425 wheat plants m^2^. Row spacing for the intercropped wheat was 0.13 m.

Wheat (cv. Xindong 20) was sown on 3 October 2014, and harvested on 10 June 2015. The jujube trees with different tree ages were pruned annually. All fields were fertilized with urea, triple superphosphate, potassium sulfate, and farmyard manure (0.37% N, 0.41% P_2_O_5_, 0.46% K_2_O) at concentrations of 15 × 10^3^ kg ha^−1^ (farmyard manure), 275 kg ha^−1^ (N), 150 kg ha^−1^ (P_2_O_5_), and 275 kg ha^−1^ (K_2_O). All farmyard manure, the P and K fertilizers, and 40% of the N fertilizer were applied evenly across the soil surface and then incorporated into the soil at a depth of 0–20 cm before the wheat was sown; the remaining 60% of the N fertilizer was spread when the wheat plants reached the stem elongation stage. The plots were irrigated three times across the whole growth period, with irrigation coinciding with the reviving, jointing, and filling stages of wheat growth. Each irrigation application included 90–100 mm (900–1,000 m^3^ ha^−1^).

The soils were sampled during the wheat filling stage, on May 20, 2015. After the removal of any crop residue, five soil cores were collected with an auger in each plot to a soil depth of 20 cm (5 cm diameter). Triplicate samples were collected. The sampling sites were 1.5 m away from the jujube trees. Immediately after their collection, the soil samples were transported on ice to the laboratory, where they were passed through a 2.0 mm mesh and divided into two subsamples. One subsample was stored at 4°C and later used to determine soil physicochemical properties, and the other subsample was stored at −20°C until it was used for DNA analysis. Ten wheat plants, five per transect (two transects in which jujube tree and wheat interact with each other), were excavated with a fork spade at each plot. The shoots were cut off at a height of ~ 5 cm, and all roots of a specific site were pooled in a plastic bag for subsequent processing. At the same time, 1-year-old young roots of jujube tree near the wheat plants were also cut off with a shovel and placed on ice in a cooler for transfer to the laboratory.

### Soil parameter measurements

Soil parameters were measured according to standard methods as described in Page et al. (1982). Soil moisture (SM) was measured by drying fresh soil samples at 105°C to constant weight. Soil pH was measured in a soil:water (1:5) extract with a pH meter. Soil organic matter (SOM) and total nitrogen (TN) were measured using the Walkley–Black and Kjeldahl method. Soil total phosphorus (TP) was measured by colorimetric analysis after digestion with sulfuric acid and perchloric acid. Soil inorganic nitrogen was measured using a continuous flow analyzer (AutoAnalyzer-AA3, Seal Analytical, Norderstedt, Germany) after extraction with 2 mol L^−1^ KCl. Soil available phosphorous (AP) was determined by the Olsen method. Soil available potassium (AK) was extracted with neutral ammonium acetate and measured by atomic absorption spectrometry (ZL-5100, PerkinElmer, MA). Soil total potassium (TK) was measured by hydrofluoric acid digestion.

### Phospholipid fatty acid

The total microbial biomass was the sum of all the biomarkers’ values. The PLFAs i13:0, i14:0, a15:0, i15:0, i16:0, a17:0, i17:0, i18:0, i19:0, and a19:0 were used as the biomarkers for Gram-positive bacteria (G+), and 17:1 ω8c, 16:1 ω11c, 15:1 ω6c, 20:1 ω9c, 3OH 12:0, 2OH 14:0, i17:0 3OH, 2OH 16:0, i17:0 3OH, cy17:0, and cy19:0 ω8c were identified as the biomarker of Gram-negative bacteria (G-). Fungi were represented by 18:1 ω9c, 18:1 ω5c, 18:3 ω6c (6, 9, 12), 20:1 ω9c, and 16:1 ω5c. 10Me 17:0 and 10Me 18:0 were used to indicate Actinomycete (ACT)-derived fatty acids; 20:1 ω9c and 16:1 ω5c were indicative of Arbuscular mycorrhizal fungi (AMF); PLFA biomarker 20:4 ω6,9,12,15c was used to identify Protozoan-derived fatty acid. General bacteria were assessed by 12:0, 14:0, 15:0, 16:0, 17:0, i17:0 w5c, and 20:0 (Frostegård et al., 2011; Zhang et al., 2019a). The bacterial biomass of PLFA was the sum of the G+, G–, and General bacteria values. The ratio of different microbial PLFA values can represent the dynamic changes between microbial groups (Fanin et al., 2019). Saturated fatty acids included PLFAs12:0, 14:0, 15:0, 16:0, 17:0, and 20:0; PLFAs biomarkers i13:0, i14:0, i15:0, a15:0, i16:0, i17:0, a17:0, i18:0, i19:0, and a19:0 were included in Methyl-branched fatty acids. In this study, the ratios of fungi to bacteria (F/B), Gram-negative bacteria to Gram-positive bacteria (G−/G+), and Saturated fatty acids to monounsaturated fatty acids (S/M) were calculated to explain relative changes of microbial biomass and community to environmental change (Frostegård et al., 2011).

### DNA extraction, PCR, and Illumina sequencing

The genomic DNA was extracted from 0.5 g of soil and 0.3 g wheat and jujube root using MoBio PowerSoil DNA Isolation Kit (MoBio Laboratories, Carlsbad, CA, United States). Genomic DNA was amplified using a nested polymerase chain reaction (PCR) procedure. The first amplification was performed in a final volume of 20 μl with 1.0 μl template DNA, 0.5 μl of each forward and reverse primer (16 pmol μl − 1), 10 μl of PCR SuperMix (TransGen Biotech Co., China), and 8.0 μl of sterile water with primer pair GeoA2-AML2 (White et al., 1990). Amplification procedure was conducted on a thermal cycler (Bio-Rad, United States) using the following conditions: 94°C for 3 min; 30 cycles of 94°C for 30 s; 40°C for 60 s; and 72°C for 1 min of extension, followed by 72°C for 10 min. Next, successful products of the first amplification were diluted at 1:100, and then was used as template in a second PCR with primer pair NS31 (Simon et al., 1992) and AMDGR (Sato et al., 2005). PCR reactions were performed under the same aforementioned conditions. Preparation of the amplicon libraries and pyrosequencing with Roche 454 GS-FLX technology were conducted at Personalbio in Shanghai, China.

The sequences were first filtered for quality and trimmed using mothur package to remove multiple identifiers and primers (version 1.31.2). And then, cleaned sequences (10,523 reads) were clustered into operational taxonomic units (OTUs) based on 97% similarity. Representative sequences from each OTU clade were blasted against the NCBI GenBank to obtain the most similar sequences from other studies. Finally, representative sequences for each OTU, blasted published sequences in the NCBI GenBank which were highly affiliated to each OTU, and representative sequences of major families of *Glomeromycotina* were used to construct a maximum likelihood tree.

### Assessment of AMF root colonization

Mycorrhizal colonization of wheat roots and jujube root was determined by the quadrant intersection method (Giovannetti and Mosse, 1980).

### Statistical analyses

All statistical analyses were performed in R 4.1.0 (R Core Team, 2020) unless otherwise noted. Indices of AMF diversity, including OTU richness, Chao1, and ACE, and of phylogenetic diversity were computed in the R package *vegan* (Oksanen et al., 2019). A principal coordinates analysis (PCoA) was performed to visualize the variation in the soil microbial and AMF communities across different tree stand ages, based on the Bray–Curtis dissimilarity. The relationships between the soil microbial and AMF communities and soil properties were determined in redundancy analyses (RDAs), also performed in the *vegan* package in R (Oksanen et al., 2019). Only environmental variables that correlated significantly (*p* < 0.05) with the RDA model were selected (calculated based on 999 permutations). Soil physicochemical parameters, AMF diversity, and the composition of the AMF community were analyzed in an ANOVA, and the least significant difference (LSD) was used to compare the means for each variable (*p* < 0.05). Co-occurrence networks of all OTUs of the AMF community were constructed based on Spearman rank correlations between OTUs to reveal significant positive correlations (*R* > 0.3 and *p* < 0.05). The results were visualized with Gephi (version 0.9.2; Li et al., 2015). A subgraph of each sample was obtained with the R package *igraph* (Csardi and Nepusz, 2006). This package was also used to calculate the topological network properties of each sample, including the total number of network nodes (representing OTUs), the total number of edges (connections between nodes representing significant positive correlations between OTUs), and the degree of co-occurrence (the number of direct correlations to a node). An ordinary least squares (OLS) linear regression model was used to test the relationship between topological network properties and wheat yield. The complex effects of abiotic and biotic factors on wheat yield were quantified by structural equation modeling (SEM) using AMOS 17.0 (SPSS, Chicago, IL, United States). Variables in the model included soil moisture, available phosphorus content, wheat yield, the proportion of Gram-positive (G+) to Gram-negative (G–) bacteria, and the degree of co-occurrence network (average degree). Maximum likelihood estimation was used to fit the covariance matrix to the model. The *a priori* theoretical model was adjusted according to the principle of the lowest chi-square, nonsignificant probability (*p* > 0.05), a high goodness-of-fit index (> 0.90), and root mean square error of approximation < 0.05 to ensure that the final model was adequately fitted (Grace and Keeley, 2006).

## Results

### Soil parameters, wheat yield, and aboveground biomass

Large differences were found in the physicochemical parameters of the soil across the three stands in the agroforestry system (Supplementary Table S2). Soil moisture, organic matter content, and available phosphorus content differed significantly with tree age and were highest in the soil of the IN3 treatment and lowest in the soil of the IN13 treatment. Total phosphorus and total potassium contents were highest in the IN3 treatment but did not differ significantly between the IN8 and IN13 treatments. Conversely, inorganic nitrogen content was highest in the IN13 treatment but did not differ significantly between the IN3 and IN8 treatments. There were no significant differences in soil pH, total nitrogen content, or available potassium content among the three treatments.

Wheat yield and aboveground biomass differed significantly among treatments (Supplementary Table S3) and decreased significantly with increasing stand age. Consequently, wheat yield and aboveground biomass were highest in IN3 and lowest in IN13.

### Soil microbial community

The Shannon diversity and Simpson diversity indices were significantly higher in IN8 and IN13 than in IN3 and were lowest in IN3 (Supplementary Table S4). The phospholipid fatty acid (PLFA) contents of the total and grouped soil microorganisms in the different treatments are shown in Table 1. PLFA diversity, which is used to characterize soil microbial communities, differed significantly among the three treatments. The total PLFA content was highest in IN8 and lowest in IN13. The trends in fungal biomass were similar to those of the total PLFA content, whereas AMF and protozoal biomass were lowest in IN13, and the differences between IN3 and IN8 were not significant. By contrast, the actinomycete content was lower in IN3 than in the other treatments. Bacteria accounted for a large proportion of the total PLFA content. The ratios of G+ to G– species and saturated to monounsaturated PLFA (S/M) showed a gradual upward trend from IN3 to IN13.

**Table 1:** Soil microorganism PLFA contents (nmol/g soil) across different tree ages. The same lowercase letters did not significantly differ among treatments at *P* < 0.05 according to LSD test.

	Total PLFA	G+	G–	Bacterial	Fungi	Actinomycetes	AMF	Protozoan	G+/G–	F/B	S/M
IN3	32.37 ± 1.31b	6.77 ± 0.90a	4.96 ± 0.57a	23.02 ± 2.04a	3.90 ± 0.52ab	0.51 ± 0.06b	1.48 ± 0.08a	0.10 ± 0.00a	1.71 ± 0.09b	0.17 ± 0.01a	1.41 ± 0.09b
IN8	38.87 ± 1.21a	7.80 ± 0.28a	4.88 ± 0.26a	26.13 ± 0.91a	4.52 ± 0.24a	0.92 ± 0.10a	1.47 ± 0.07a	0.10 ± 0.00a	2.19 ± 0.06a	0.17 ± 0.01a	1.65 ± 0.07b
IN13	31.63 ± 1.83b	6.62 ± 0.53a	3.90 ± 0.38a	21.09 ± 2.46a	2.81 ± 0.34b	0.58 ± 0.05b	0.96 ± 0.06b	0.08 ± 0.01b	2.27 ± 0.06a	0.13 ± 0.00b	1.94 ± 0.05a

**Figure 1:**
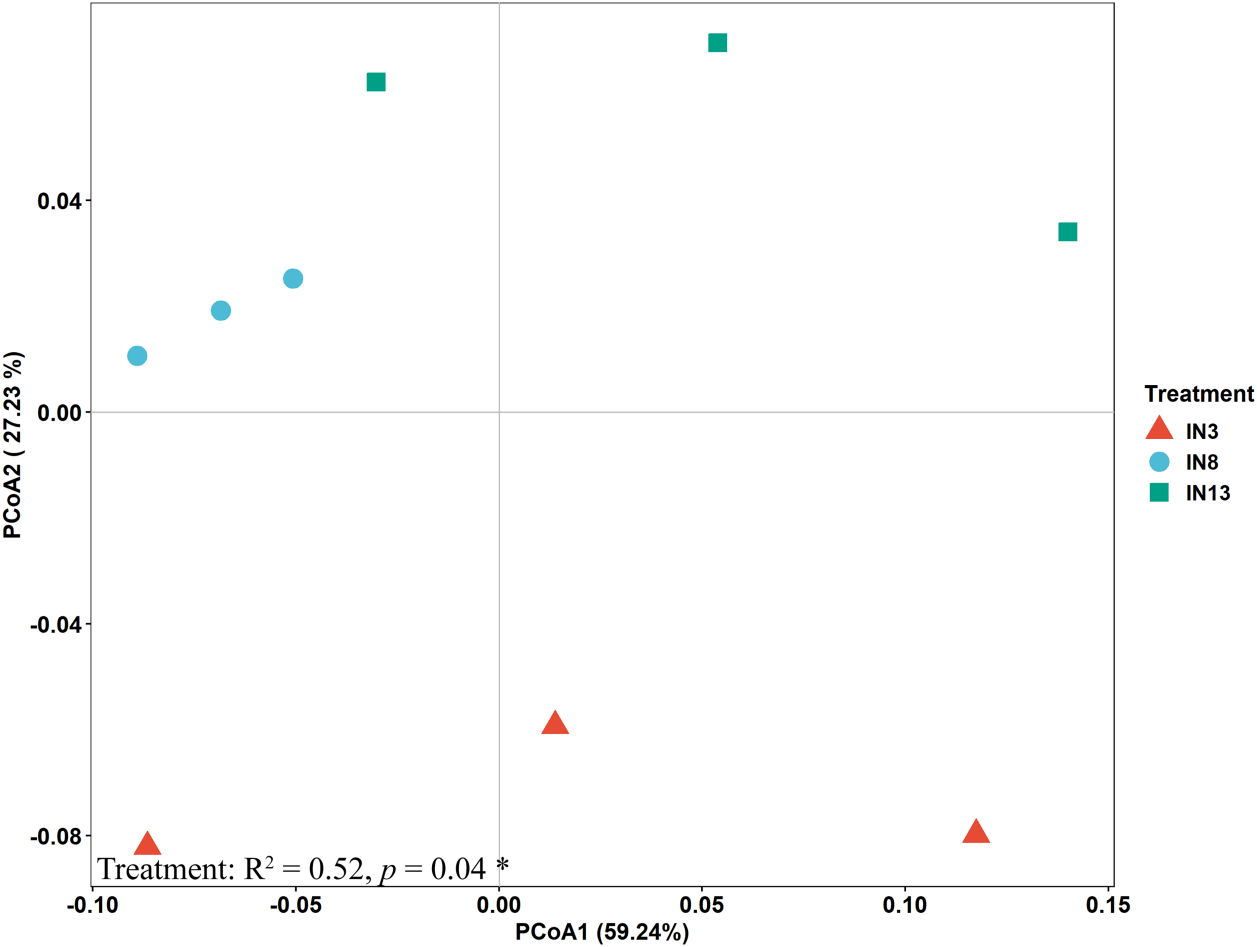
Principal coordinates analysis (PCoA) of soil microbial community based on the Bray–Curtis dissimilarities under different tree ages of intercropping systems.

The PCoA provided further evidence that the soil microbial community was significantly distinct across the three stand ages (Figure 1). Three distinct clusters in the ordination graph were seen, with PCoA1 explaining 59.24% of the variance and PCoA2 explaining 27.23%. The PERMANOVA also showed significant effects of tree age and cropping system on soil microbial communities. A CCA was performed to determine the relationship between soil parameters and the microbial community. Soil moisture (*R*^2^ = 0.87, *p* < 0.01), total phosphorus (*R*^2^ = 0.79, *p* = 0.01), and available phosphorus (*R*^2^ = 0.66, *p* = 0.05) had significant effects on the soil microbial community (Supplementary Figure S2A).

### Composition of the AMF community

**Figure 2:**
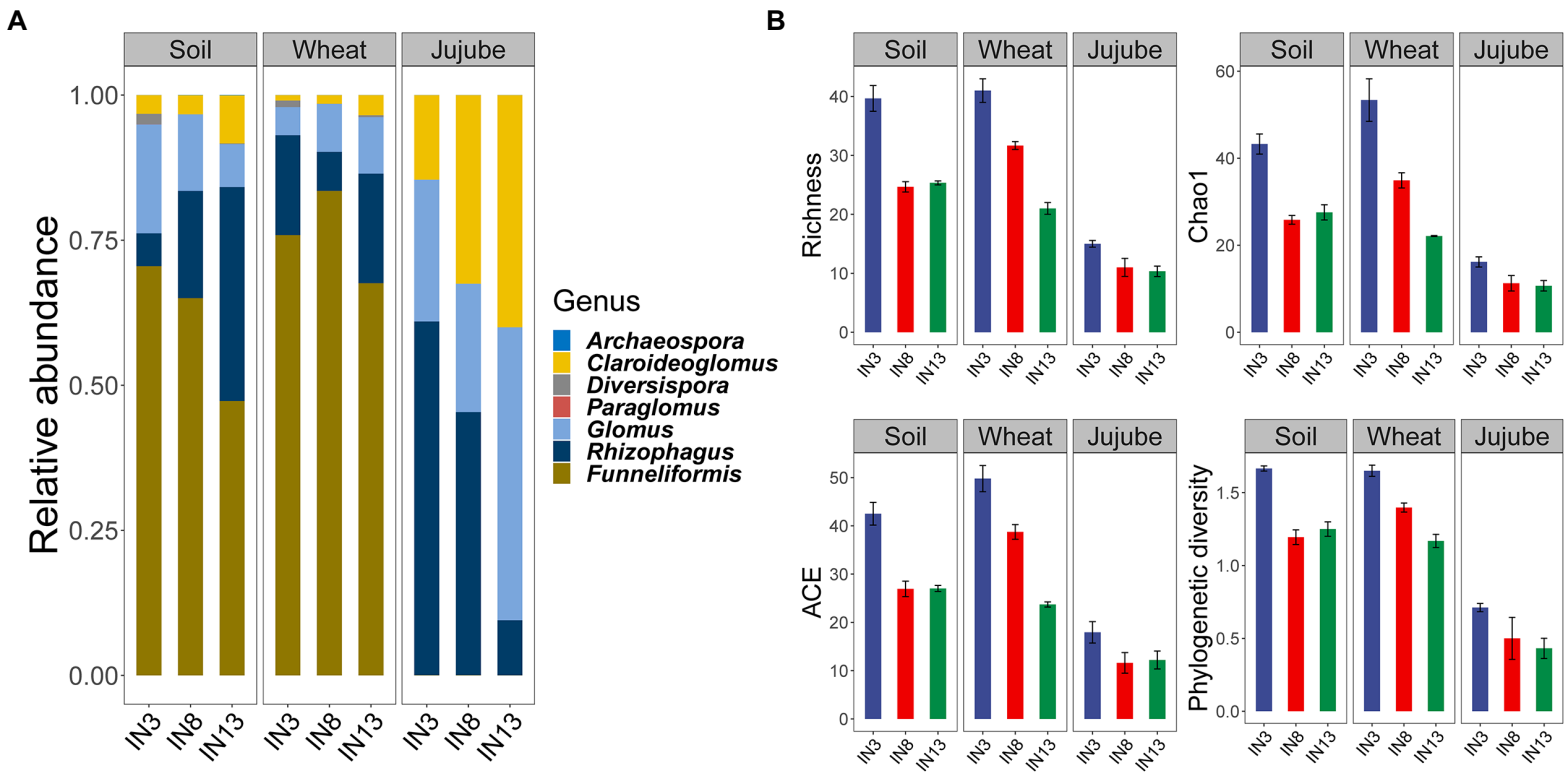
Relative abundance **(A)**, and α diversity **(B)**, of arbuscular mycorrhizal fungi in soil, wheat root and jujube root under different tree ages.

For the AMF, 68 OTUs representing seven genera were obtained. The two most abundant genera in soil and wheat root were *Funneliformis* (60.92 and 75.64%) and *Rhizophagus* (20.37 and 14.30%), whereas the most two abundant genera in jujube root were *Rhizophagus* (38.36%) and *Glomus* (32.35%; Figure 2A). In the soil AMF community, the relative abundances of *Diversispora*, *Funneliformis*, *Glomus*, and *Paraglomus* were significantly higher in IN3 than in IN8 or IN13, whereas the relative abundances of *Claroideoglomus* and *Rhizophagus* were much higher in IN13 than in IN3 or IN8 (Supplementary Figure S3). In the wheat root AMF community, the relative abundances of *Funneliformis* and *Rhizophagus* were much higher in IN8, and the abundances of *Diversispora* and *Claroideoglomus* were significantly higher in IN3 and IN13, respectively (Supplementary Figure S3). In jujube root, the relative abundance of *Rhizophagus* was significantly higher in IN3, that of *Claroideoglomus* was significantly higher in IN8, and that of *Glomus* was significantly higher in IN13 (Supplementary Figure S3). The different treatments in the different niches also resulted in significantly distinct AMF diversity (Figure 2B). In soil and jujube root, richness, Chao1, ACE, and phylogenetic diversity were significantly higher in IN3 than in IN8 and IN13, although the differences between the latter two stands were not significant. However, all diversity indices showed a downward trend in jujube trees/wheat intercropping systems of increasing stand age (Figure 2B).

**Figure 3:**
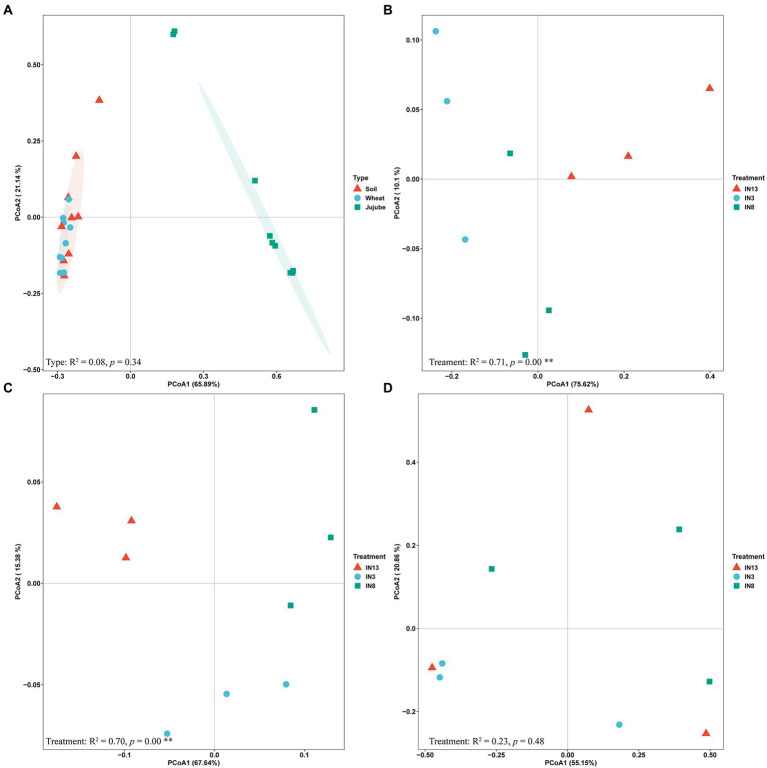
Principal coordinates analysis (PCoA) of arbuscular mycorrhizal fungi community based on the Bray–Curtis dissimilarities in different niches **(A)** and tree ages as well as cropping system of soil **(B)**, jujube **(C)** and wheat **(D)**.

According to the PCoA, the AMF community did not differ significantly between soil and wheat root, whereas that in jujube root formed distinct clusters. PCoA1 and PCoA2 explained 65.89 and 21.14% of the variance, respectively (Figure 3A). The composition of the AMF communities in soil and wheat root varied significantly across the three stand ages, with PCoA1 explaining 75.82 and 67.64% of the variance and PCoA2 explaining 10.10 and 10.38%, respectively (Figures 3B, C). By contrast, the composition of the AMF community of jujube root did not differ significantly across the three stand ages (Figure 3D).

The relationship between soil parameters and the AMF community was assessed in a CCA (Supplementary Figure S2). Significant effects on the AMF communities of soil and wheat root were found for total phosphorus (*R*^2^ = 0.77, *p* = 0.02; *R*^2^ = 0.71, *p* = 0.03), soil moisture (*R*^2^ = 0.74, *p* = 0.02; *R*^2^ = 0.75, *p* = 0.02), inorganic nitrogen (*R*^2^ = 0.71, *p* = 0.03; *R*^2^ = 0.85, *p* = 0.01), and available phosphorus (*R*^2^ = 0.67, *p* = 0.04; *R*^2^ = 0.89, *p* < 0.01; Supplementary Figures S2B,C). In addition, soil organic matter had a significant effect on the AMF community of wheat root, and available potassium and total potassium had a significant effect on the AMF community of jujube root (Supplementary Figure S2D).

### Co-occurrence networks of AMF

**Figure 4:**
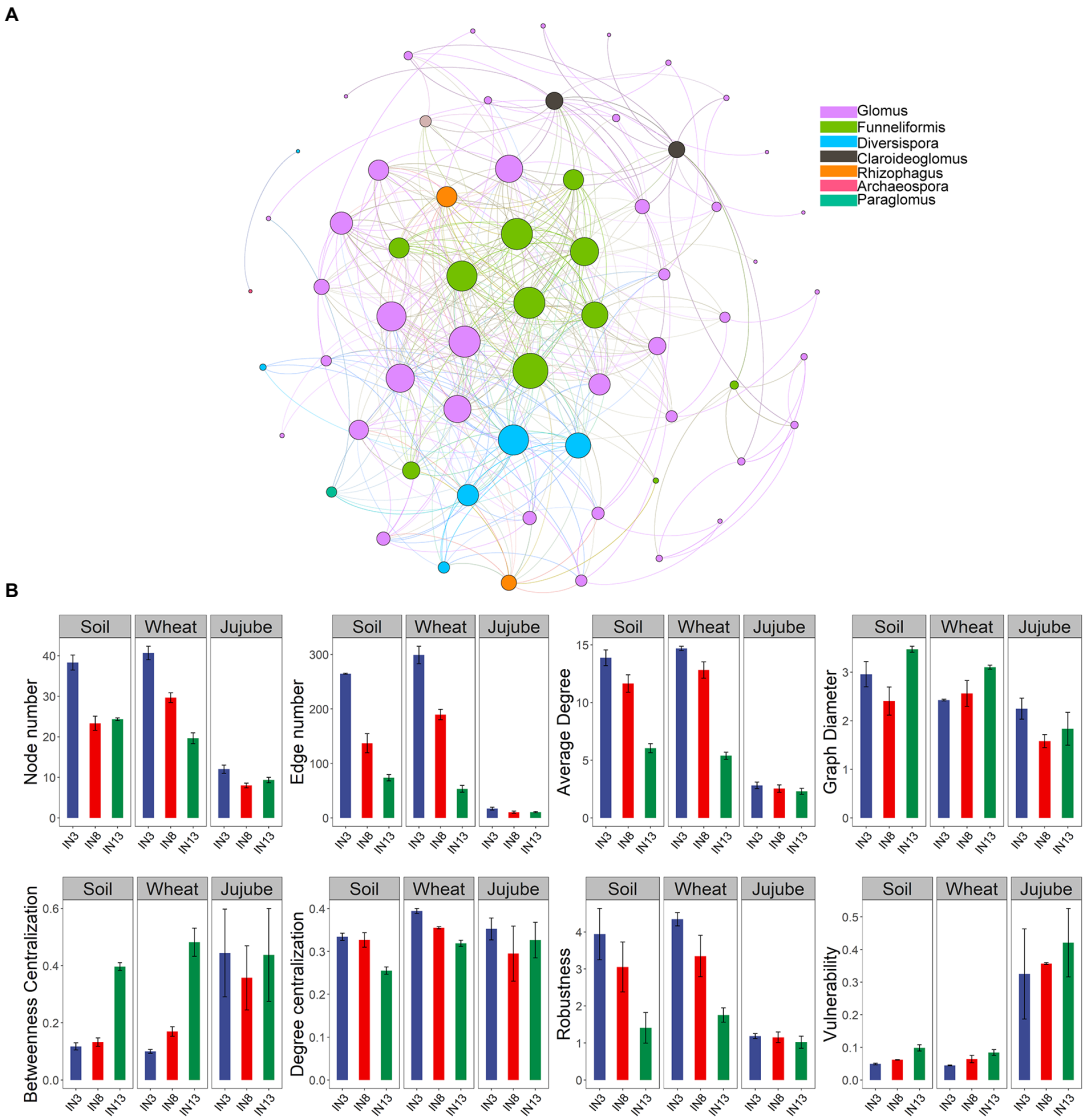
Co-occurrence network of arbuscular mycorrhizal fungi **(A)**. Topological parameters of arbuscular mycorrhizal fungal co-occurrence network in soil, wheat root and jujube root under different tree ages **(B)**.

Co-occurrence networks were constructed to investigate the effects of stand age on AMF interactions. A total of 68 nodes and 388 edges were detected (Figure 4). A subgraph of each sample was extracted, and the topological network parameters of node and edge numbers, average degree, graph diameter, betweenness centralization, degree centralization, robustness, and vulnerability were calculated to assess the complexity and stability of the AMF network across the different treatments. Larger node and edge numbers, average degree, and degree centralization and smaller betweenness centralization represent greater network complexity. Higher robustness and lower vulnerability indicate greater network stability. For soil and wheat root, node and edge numbers, average degree, degree centralization, and robustness were highest in the IN3 treatment, which indicates its greater network complexity and stability (Figure 4B). Network complexity and stability showed a downward trend from IN3 to IN13 in wheat root (Figure 4B). These results strongly suggest that older stand age negatively affects AMF associations and reduces the complexity and stability of AMF community networks.

### Relationship of soil microbial community, AMF diversity, and complexity and stability of co-occurrence networks to wheat yield and aboveground biomass

**Figure 5:**
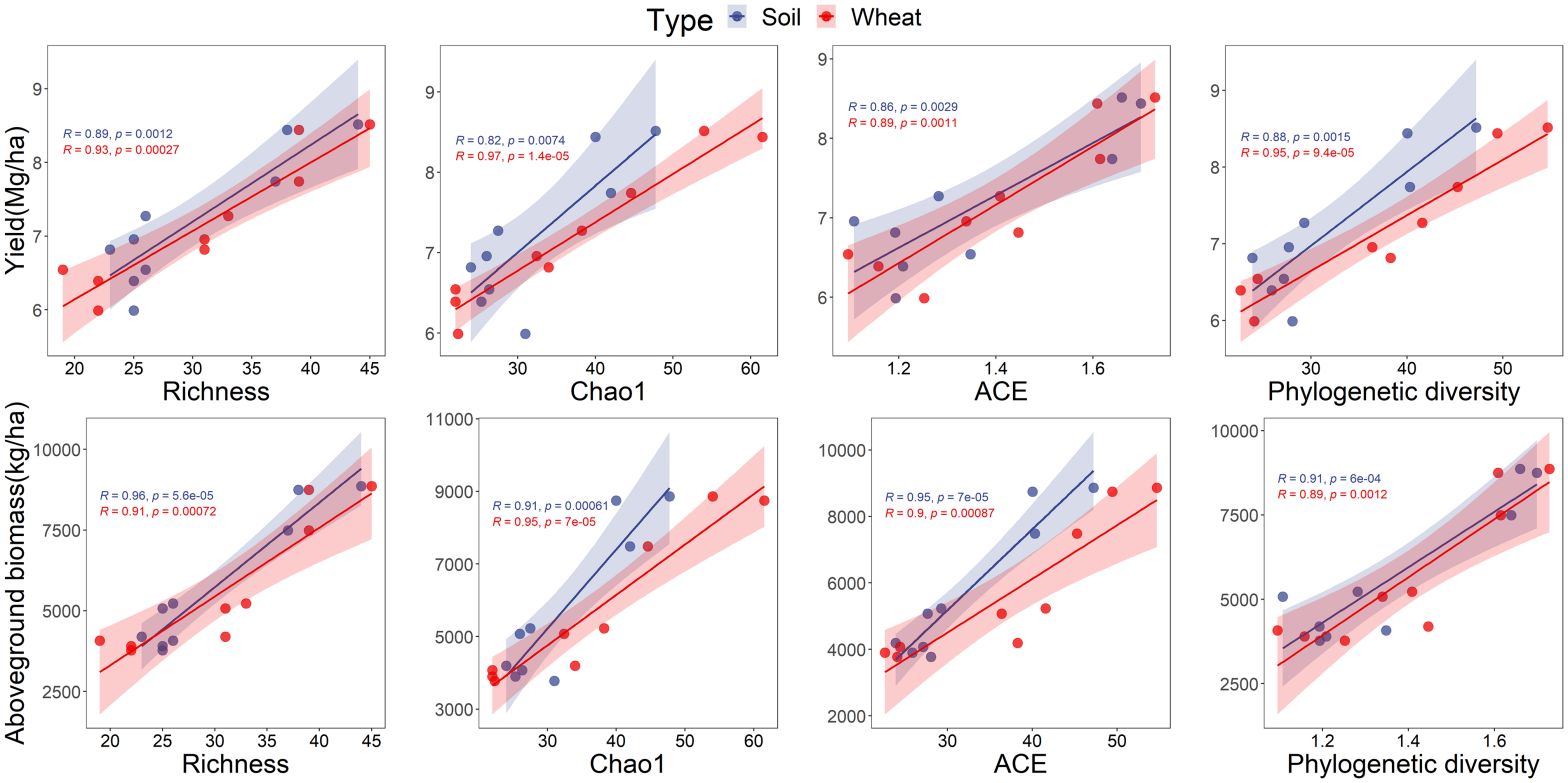
The relationships of wheat yield, aboveground biomass and α diversity of arbuscular mycorrhizal fungi.

**Figure 6:**
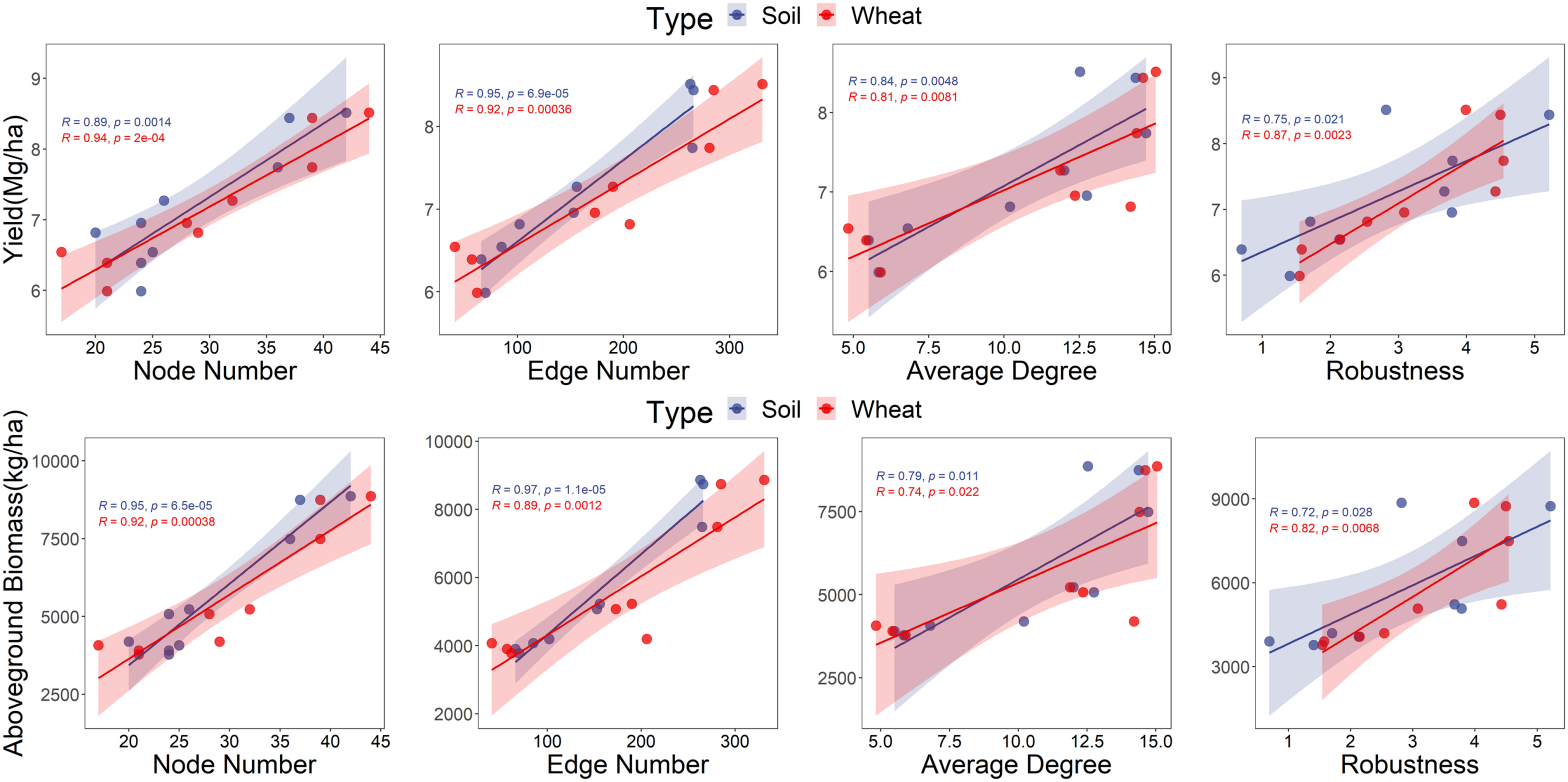
The relationships of wheat yield, aboveground biomass and topological parameters of arbuscular mycorrhizal fungi.

Positive and negative relationships were found between grouped PLFA contents, AMF diversity, network parameters, and both wheat yield and aboveground biomass. For example, the AMF content of the soil microbial community was positively related to wheat yield (Table 2), and both G+/G– and S/M were negatively related to wheat yield and aboveground biomass (Table 2). Wheat yield and aboveground biomass responded positively to the relative abundance of *Diversispora* and negatively to the relative abundance of *Rhizophagus* in the soil AMF community (Table 3). Positive responses of wheat yield and aboveground biomass to the relative abundances of *Funneliformis* and *Glomus*, respectively, were also found (Table 3). For the AMF community of wheat root, the relative abundance of *Rhizophagus* was related to wheat yield and aboveground biomass, whereas the relative abundance of *Claroideoglomus* responded negatively to wheat yield (Table 3). Significant positive relationships were found between soil AMF diversity (richness, Chao1, ACE, phylogenetic diversity) and wheat yield (Figure 5), and between soil AMF network complexity and stability and both wheat yield and aboveground biomass, which indicated that interactions between AMF communities promoted wheat growth (Figure 6).

**Table 2:** Pearson correlations between grouped PLFA contents and wheat yield as well as aboveground biomass.

PLFA contents	Yield		Aboveground biomass	
	*R* ^2^	*p*	*R* ^2^	*p*
Total PLFA	0.04	0.63	0.03	0.64
G+	0.07	0.48	0.07	0.49
G–	0.07	0.51	0.08	0.46
Bacterial	0.01	0.8	0.01	0.8
Fungi	0.04	0.59	0.03	0.69
Actinomycetes	0.09	0.43	0.22	0.21
AMF	**0.48**	**0.04** ^*^	0.4	0.06
Protozoan	0.36	0.08	0.31	0.12
G+/G–	**0.83**	**<0.01** ^**^	**0.9**	**<0.01** ^**^
F/B	0.28	0.14	0.19	0.23
S/M	**0.66**	**<0.01** ^**^	**0.59**	**0.02** ^*^

Value in bold indicates a significant difference. ^**^indicates an extremely significant difference at *p* < 0.01, ^*^indicates a significant difference at *p* < 0.05.

**Figure 7:**
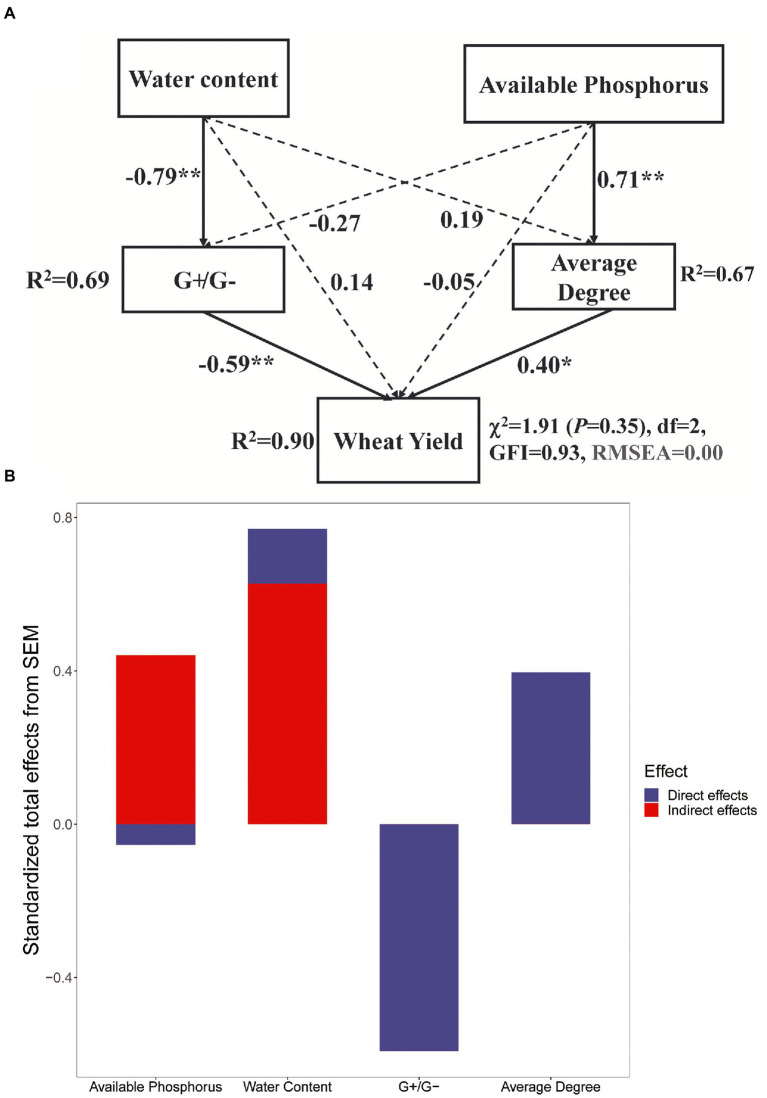
Effects of abiotic and biotic factors on wheat yield **(A)**. ^*^indicates *p* < 0.05; ^**^indicates *p* < 0.01. Continuous and dashed lines indicate significant and non-significant relationships, respectively. *R*^2^ denotes the proportion of variance explained. **(B)** Standardized total effects (direct plus indirect effects) derived from the structural equation models depicted above.

Structural equation modeling was performed to examine the hypothesized direct and indirect relationships between soil physicochemical parameters (soil moisture, available phosphorus), soil microbial community resistance (G+/G–), AMF network complexity (average degree), and wheat yield. The model explained 94.0% of the variance in wheat yield (Figure 7A). It also showed that stand age and G+/G– had negative indirect and direct effects, respectively, on wheat yield. Positive effects on wheat yield were found for soil moisture, available phosphorus content, and soil AMF network complexity (Figure 7B).

**Table 3:** Pearson correlations between relative abundance of AM fungi on genus level and wheat yield as well as aboveground biomass.

	Soil				Wheat root			
	Yield		Aboveground biomass		Yield		Aboveground biomass	
	*R* ^2^	*p*	*R* ^2^	*p*	*R* ^2^	*p*	*R* ^2^	*p*
*Archaeospora*	−0.39	0.3	−0.44	0.24	–	–	–	–
*Claroideoglomus*	−0.63	0.07	−0.6	0.09	**−0.66**	**0.05** ^*^	−0.64	0.07
*Diversispora*	**0.77**	**0.02** ^*^	**0.81**	**0.01** ^*^	–	–	–	–
*Funneliformis*	**0.74**	**0.02** ^*^	0.62	0.08	0.59	0.09	0.59	0.1
*Glomus*	0.64	0.06	**0.68**	**0.05** ^*^	−0.65	0.06	−0.53	0.14
*Paraglomus*	0.54	0.13	0.62	0.07	–	–	–	–
*Rhizophagus*	**−0.88**	**0.00** ^**^	**−0.8**	**0.01** ^*^	**−0.88**	**0.00** ^**^	**−0.8**	**0.01** ^*^

Value in bold indicates a significant difference.

^**^indicates an extremely significant difference at *p* < 0.01,

^*^indicates a significant difference at *p* < 0.05.

## Discussion

### The effects of stand age on the soil microbial community

Soil communities are extremely diverse (Robeson et al., 2011), as are their interactions with plants in supporting their growth (Compant et al., 2010; van Dam and Bouwmeester, 2016). In this study, the microbial communities of jujube stand of different ages in an agroforestry system were remarkably distinct (Figure 1), which supports our first hypothesis that soil microbial community could be affected by tree ages. Subsets of the microbial community can be defined based on specific microbial groups to identify variation in composition (Yao et al., 2016; Hugerth and Andersson, 2017). The total PLFA content, as well as the bacterial, G+, fungal, and actinomycete contents, was significantly higher in IN8 than in IN3 or IN13 (Table 1). Consistent with this result, the diversity of the soil microbial community was also highest in the IN8 treatment (Supplementary Table S4).

However, we also found that different groups of soil microorganisms responded differently to the increase in stand age. Thus, G+ was highest in IN8 and lowest in IN13, whereas G– showed a downward trend, differences that might have been due to different microbial life strategies and changes in soil parameters (Jing et al., 2019). Soil moisture is a major driver of the soil microbial community in agroforestry systems (Radhakrishnan and Varadharajan, 2016; Beule et al., 2020). The deeper soil water consumption by the roots of older trees significantly reduces water recharge to the soil and surface water, thus undermining the water supply available for crop growth (Ward et al., 2002). In addition, G+ communities are more resistant to drying/rewetting than G– communities due to their physiological characteristics such as the presence of a strong, thick, interlinked peptidoglycan cell wall (Schimel et al., 2007). Indeed, the G+/G– ratio has been recognized as a critical indicator to indicate resistance of microbial communities to perturbations (De Vries and Shade, 2013). A shift toward a greater presence of G+ (higher values of G+/G–) can be viewed as a mechanism allowing adaptation to a semi-arid climate as well as an indicator of a gradual change from copiotrophic to more oligotrophic conditions (Yao et al., 2006; Bastida et al., 2015). This may explain the upward trend in G+/G– with increasing stand age, and the significant negative relationship with soil moisture and soil phosphorus contents.

Significant variation in fungal biomass with tree growth was also found (Table 1). The contribution of fungi to the degradation of more recalcitrant material is larger than that of bacteria (Boer et al., 2005), such that the increase in fungal biomass in IN8 can be attributed to the competitive advantage to fungi conferred by the presence of stabilized substrates. The dramatic decline in fungal biomass in IN13 may have been due to adverse soil conditions, as a decline in soil moisture, soil nutrient, and organic matter adversely affect microbial growth (Bell et al., 2008; Köster et al., 2014). In fungi, the large amounts of energy needed to tolerate drought and low resource availability lead to a decline in fungal growth (Kempf and Bremer, 1998; Oren, 2008).

### Variation in the AMF community across tree stands of different ages

The AMF community varied significantly among different niches (Figures 2, 3A). Although there is no host specificity between AM fungi and plants, different AM fungi have certain preferences for host plants (Campos et al., 2018). Plant species function as biotic filters, based on their preferences for specific AMF species (Kiers et al., 2011; Torrecillas et al., 2012). The AMF communities of soil and wheat were more similar than those of soil and jujube root (Figure 3). A possible explanation for this is that tillage before wheat planting destroyed jujube roots, and jujube, as a perennial plant, has slower root growth than wheat (Hailemariam et al., 2013). AMF obtain carbon from host plants and rely on plant photosynthetic capacity and the translocation of photosynthate to the root to meet their carbon needs (Shukla et al., 2009).

The significant changes in the community as a function of tree age further support our first hypothesis (Figures 2, 3). The downward trend in AMF diversity from IN3 to IN13 can be attributed to changes in the soil environment. In soil and in wheat root, community variation was induced by soil moisture as well as available phosphorus and inorganic nitrogen. Neither phosphorus nor nitrogen was lacking in the soil, which showed a negative relationship with AMF diversity, in contrast to soil moisture. Therefore, the absence of soil water would lead to the extinction of several species of AMF. Previous studies have shown that soil moisture acts as an abiotic filter that affects AMF community assembly by regulating AMF colonization and phylotype diversity (Deepika and Kothamasi, 2015). In jujube root, changes over time would include progressive lignification with increasing stand age, such that colonization by AMF would be increasingly challenging (Sheng et al., 2017), as evidenced by the observed changes in mycorrhizal colonization among the three jujube stands.

As the environment changed across the different treatments, so did the AMF communities colonizing the plant roots (Supplementary Figures S2, S3). AMF taxa can be classified according to their suitability to specific habitats, and their relative abundance in soil depends on the availability of suitable habitats and favorable host plants (Verbruggen et al., 2012). Different AMF taxa respond differently to variation in the abiotic environment (Sheng et al., 2017; Liu et al., 2019; Marro et al., 2022). For example, *Acaulosporaceae* and *Gigasporaceae* are more tolerant of acidic soil environments than most *Glomeraceae* (Veresoglou et al., 2013). However, some studies have shown that *Gigasporales* are sensitive to increased land-use intensity or disturbance, while *Glomerales* remain mostly unaffected under these conditions (Marro et al., 2022). Consistent with our results, Sheng et al. (2017) reported that a root-colonizing AMF community varied with stand age. In our study, we identified several significant relationships between soil parameters and the relative abundance of AMF at the genus level (Supplementary Figure S4). The relative abundance of *Glomus* was negatively related to soil moisture, whereas that of *Diversispora* was positively related to it in wheat root. These differences may have been due to the life strategies of different root-colonizing species of AMF (Sýkorová et al., 2007). *Glomus* species are often recognized as competitive root colonizers because they are able to colonize roots from spores (Herrmann et al., 2016), which could explain the increase in their relative abundance with increasing tree age. *Diversispora* prefer well-watered conditions (Cheng et al., 2021). *Rhizophagus*, a highly infective taxon and prolific producer of vesicles in roots, prefers roots (Souza, 2015; Knegt et al., 2016), which may explain its increased relative abundance in soil and wheat.

### The contribution of AMF to wheat yield in agroforestry systems

Crop root systems have an inherent capability to adjust to complex soil environmental conditions (Malamy, 2005), including the secretion of a large array of primary or secondary plant metabolites into the soil to facilitate interactions with the biotic and abiotic environment (van Dam and Bouwmeester, 2016). In turn, host-specific changes in microbial composition feedback on plant fitness (Bever, 2003). In this study, SEM suggested that the changes in soil parameters induced by tree age led to variation in the composition of soil microbial and AMF communities and therefore functional changes that ultimately affect crop growth (Figure 6). In our study, changes in soil moisture resulted in changes in the bacterial community, and available phosphorus had a positive effect on AMF network complexity. Changes in G+/G– and AMF network complexity had negative and positive effects, respectively, on wheat yield. The negative effects of bacteria on crop yield may reflect functional trade-offs between stress tolerance and the promotion of nutrient cycling. A previous study found an inverse relationship between the stability of the microbial community and the resistance of microbial biomass and activity (Piton et al., 2021). Therefore, a reorganization of the microbial community would promote ecosystem stability through functional compensation among species responding to environmental change (Allison and Martiny, 2008; Jurburg et al., 2017).

We also found that AMF content, diversity, and network complexity responded positively to wheat yield (Table 2), which supports our second hypothesis. AMF promote host plant growth by supplying nutrients, in particular N and P (Menezes et al., 2016; Guzman et al., 2021), and enhance the tolerance of plants to various stresses, such as drought and high temperature (Duc et al., 2018; Begum et al., 2019). The extracellular hyphae of AMF can facilitate the absorption and utilization of water by plants, which is important to preventing drought damage in plants (Bahadur et al., 2019). Other studies have found that drought resistance and the better performance of crops can be attributed to the accumulation of antioxidant enzymes (superoxide dismutase, peroxidase, and catalase) and soluble sugar produced by the AMF symbiosis (Zhang et al., 2019b). Besides, the lower colonization of AMF in older jujube root due to the lignification limited their diversity as well as the faster establishment of root colonization when switching from one crop to another (Mason and Wilson, 1994). Our results support the indispensable roles of soil microorganisms, in particular AMF, in promoting plant growth in agroforestry systems.

## Conclusion

Soil microbial and AMF communities are significantly affected by the stand ages of trees in an agroforestry system. Soil moisture and the available phosphorus content related to tree age are the major drivers of these communities, which, in turn, affect crop growth. Our results also showed that AMF contribute to crop growth in agroforestry and are predictors of plant growth in agroforestry systems. However, according to a network analysis, AMF diversity and network complexity decrease with increasing stand age. Thus, stand age as well as the trade-offs among soil function, productivity, biodiversity, and economic benefits must be taken into account when establishing an agroforestry system.

## Data availability statement

The data presented in the study are deposited in the NCBI repository, accession number PRJNA887214.

## Author contributions

XQ, LHX, JL, LX, and HZ conducted the experiments. TS, JJ, and SB analyzed the data. XQ and HZ coordinated the long-term field experiments. XQ, TS, JJ, and SB wrote the manuscript. All authors contributed to the article and approved the submitted version.

## Funding

This work was funded by the Key Cultivation Project of Scientific and Technological Innovation of Xinjiang Academy of Agricultural Sciences (xjkcpy-003), Key Research and Development Projects of Xinjiang (2021B02002), National Natural Science Foundation of China (Grant Nos. 31560587, 32160521, 32060433), The central government guides local funds for science and technology development (2060503), and the Modern Agricultural Industry Technology System (CARS-03-49).

## Conflict of interest

The authors declare that the research was conducted in the absence of any commercial or financial relationships that could be construed as a potential conflict of interest.

## Publisher’s note

All claims expressed in this article are solely those of the authors and do not necessarily represent those of their affiliated organizations, or those of the publisher, the editors and the reviewers. Any product that may be evaluated in this article, or claim that may be made by its manufacturer, is not guaranteed or endorsed by the publisher.

## References

[ref1] Allison S. D. Martiny J. B. (2008). Resistance, resilience, and redundancy in microbial communities. Proc. Natl. Acad. Sci. U. S. 105, 11512–11519. doi: 10.1073/pnas.0801925105 PMC2556421 18695234

[ref2] Araujo A. S. F. Leite L. F. C. Iwata B. F. Lira M. A. Xavier G. R. Figueiredo M. V. B. (2012). Microbiological process in agroforestry systems. A review. Agron. Sustain. Dev. 32, 215–226. doi: 10.1007/s13593-011-0026-0

[ref3] Baah-Acheamfour M. Carlyle C. N. Bork E. W. Chang S. X. (2014). Trees increase soil carbon and its stability in three agroforestry systems in Central Alberta, Canada. Forest Ecol. Manag. 328, 131–139. doi: 10.1016/j.foreco.2014.05.031

[ref4] Bagyaraj D. J. Thilagar G. Ravisha C. Kushalappa C. G. Krishnamurthy K. N. Vaast P. (2015). Below ground microbial diversity as influenced by coffee agroforestry systems in the Western Ghats, India. Agric. Ecosyst. Environ. 202, 198–202. doi: 10.1016/j.agee.2015.01.015

[ref5] Bahadur A. Batool A. Nasir F. Jiang S. Mingsen Q. Zhang Q.. (2019). Mechanistic insights into arbuscular mycorrhizal fungi-mediated drought stress tolerance in plants. Int. J. Mol. Sci. 20:4199. doi: 10.3390/ijms20174199, PMID: 31461957 PMC6747277

[ref6] Barrios E. (2007). Soil biota, ecosystem services and land productivity. Ecol. Econ. 64, 269–285. doi: 10.1016/j.ecolecon.2007.03.004

[ref7] Bastida F. Selevsek N. Torres I. F. Hernández T. García C. (2015). Soil restoration with organic amendments: linking cellular functionality and ecosystem processes. Sci. Rep. 5:15550. doi: 10.1038/srep15550, PMID: 26503516 PMC4621494

[ref8] Begum N. Qin C. Ahanger M. A. Raza S. Khan M. I. Ashraf M.. (2019). Role of arbuscular mycorrhizal fungi in plant growth regulation: implications in abiotic stress tolerance. Front. Plant Sci. 10:1068. doi: 10.3389/fpls.2019.01068, PMID: 31608075 PMC6761482

[ref9] Bell C. McIntyre N. Cox S. Tissue D. Zak J. (2008). Soil microbial responses to temporal variations of moisture and temperature in a Chihuahuan Desert grassland. Microb. Ecol. 56, 153–167. doi: 10.1007/s00248-007-9333-z, PMID: 18246293

[ref10] Beule L. Lehtsaar E. Corre M. D. Schmidt M. Veldkamp E. Karlovsky P. (2020). Poplar rows in temperate agroforestry croplands promote bacteria, fungi, and denitrification genes in soils. Front. Microbiol. 10:3108. doi: 10.3389/fmicb.2019.03108, PMID: 32038551 PMC6988714

[ref11] Bever J. D. (2003). Soil community feedback and the coexistence of competitors: conceptual frameworks and empirical tests. New Phytol. 157, 465–473. doi: 10.1046/j.1469-8137.2003.00714.x, PMID: 33873396

[ref12] Boer W. De Folman L. B. Summerbell R. C. Boddy L. (2005). Living in a fungal world: impact of fungi on soil bacterial niche development. FEMS Microbiol. Rev. 29, 795–811. doi: 10.1016/j.femsre.2004.11.005, PMID: 16102603

[ref13] Bryla D. R. Eissenstat D. M. (2005). “Respiratory costs of mycorrhizal associations,” in Plant Respiration. eds. Lambers H. Ribas-Carbo M. (Dordrecht: Springer), 207–224.

[ref14] Campos C. Carvalho M. Brígido C. Goss M. J. Nobre T. (2018). Symbiosis specificity of the preceding host plant can dominate but not obliterate the association between wheat and its arbuscular mycorrhizal fungal partners. Front. Microbiol. 9:2920. doi: 10.3389/fmicb.2018.02920, PMID: 30542338 PMC6277769

[ref15] Caravaca F. Rodriguez-Caballero G. Campoy M. Sanleandro P. M. Roldán A. (2020). The invasion of semiarid Mediterranean sites by *Nicotiana glauca* mediates temporary changes in mycorrhizal associations and a permanent decrease in rhizosphere activity. Plant Soil 450, 217–229. doi: 10.1007/s11104-020-04497-1

[ref16] Cheng X. F. Wu H. H. Zou Y. N. Wu Q. S. Kuča K. (2021). Mycorrhizal response strategies of trifoliate orange under well-watered, salt stress, and waterlogging stress by regulating leaf aquaporin expression. Plant Physiol. Biochem. 162, 27–35. doi: 10.1016/j.plaphy.2021.02.026, PMID: 33662869

[ref17] Compant S. Clément C. Sessitsch A. (2010). Plant growth-promoting bacteria in the rhizo- and endo-sphere of plants: their role, colonization, mechanisms involved and prospects for utilization. Soil Biol. Biochem. 42, 669–678. doi: 10.1016/j.soilbio.2009.11.024

[ref18] Csardi G. Nepusz T. (2006). The igraph software package for complex network research. InterJ. Complex Syst. 1695, 1–9. Available at: https://igraph.org

[ref19] De Vries F. T. Shade A. (2013). Control son soil microbial community stability under climate change. Front. Microbiol. 4, 1–16. doi: 10.3389/fmicb.2013.00265 24032030 PMC3768296

[ref20] Deepika S. Kothamasi D. (2015). Soil moisture—a regulator of arbuscular mycorrhizal fungal community assembly and symbiotic phosphorus uptake. Mycorrhiza 25, 67–75. doi: 10.1007/s00572-014-0596-1, PMID: 25085217

[ref21] Dollinger J. Jose S. (2018). Agroforestry for soil health. Agrofor. Syst. 92, 213–219. doi: 10.1007/s10457-018-0223-9

[ref22] Duc N. H. Csintalan Z. Posta K. (2018). Arbuscular mycorrhizal fungi mitigate negative effects of combined drought and heat stress on tomato plants. Plant Physiol. Biochem. 132, 297–307. doi: 10.1016/j.plaphy.2018.09.011, PMID: 30245343

[ref23] Duchene O. Vian J. F. Celette F. (2017). Intercropping with legume for agroecological cropping systems: complementarity and facilitation processes and the importance of soil microorganisms. A review. Agric. Ecosyst. Environ. 240, 148–161. doi: 10.1016/j.agee.2017.02.019

[ref24] Fanin N. Kardol P. Farrell M. Nilsson M. C. Gundale M. J. Wardle D. A. (2019). The ratio of gram-positive to gram-negative bacterial PLFA markers as an indicator of carbon availability in organic soils. Soil Biol. Biochem. 128, 111–114. doi: 10.1016/j.soilbio.2018.10.010

[ref25] Fentahun M. Hager H. (2010). Integration of indigenous wild woody perennial edible fruit bearing species in the agricultural landscapes of Amhara region, Ethiopia. Agrofor. Syst. 78, 79–95. doi: 10.1007/s10457-009-9239-5

[ref26] Frostegård Å. Tunlid A. Bååth E. (2011). Use and misuse of PLFA measurements in soils. Soil Biol. Biochem. 43, 1621–1625. doi: 10.1016/j.soilbio.2010.11.021

[ref27] Gao P. Zheng X. Wang L. Liu B. Zhang S. (2019). Changes in the soil bacterial community in a chronosequence of temperate walnut-based intercropping systems. Forests 10:299. doi: 10.3390/f10040299

[ref28] Giovannetti M. Mosse B. (1980). An evaluation of techniques for measuring vesicular arbuscular mycorrhizal infection in roots. New Phytol. 84, 489–500. doi: 10.1111/j.1469-8137.1980.tb04556.x

[ref29] Grace J. B. Keeley J. E. (2006). A structural equation model analysis of postfire plant diversity in California shrublands. Ecol. Appl. 16, 503–514. doi: 10.1890/1051-0761(2006)016[0503:ASEMAO]2.0.CO;2, PMID: 16711040

[ref30] Guzman A. Montes M. Hutchins L. DeLaCerda G. Yang P. Kakouridis A.. (2021). Crop diversity enriches arbuscular mycorrhizal fungal communities in an intensive agricultural landscape. New Phytol. 231, 447–459. doi: 10.1111/nph.17306, PMID: 33638170 PMC9292320

[ref31] Hailemariam M. Birhane E. Asfaw Z. Zewdie S. (2013). Arbuscular mycorrhizal association of indigenous agroforestry tree species and their infective potential with maize in the rift valley, Ethiopia. Agrofor. Syst. 87, 1261–1272. doi: 10.1007/s10457-013-9634-9

[ref32] Herrmann L. Lesueur D. Bräu L. Davison J. Jairus T. Robain H.. (2016). Diversity of root-associated arbuscular mycorrhizal fungal communities in a rubber tree plantation chronosequence in Northeast Thailand. Mycorrhiza 26, 863–877. doi: 10.1007/s00572-016-0720-5, PMID: 27448680

[ref33] Hugerth L. W. Andersson A. F. (2017). Analysing microbial community composition through amplicon sequencing: from sampling to hypothesis testing. Front. Microbiol. 8:1561. doi: 10.3389/fmicb.2017.01561, PMID: 28928718 PMC5591341

[ref34] Hutchins D. A. Jansson J. K. Remais J. V. Rich V. I. Singh B. K. Trivedi P. (2019). Climate change microbiology—problems and perspectives. Nat. Rev. Microbiol. 17, 391–396. doi: 10.1038/s41579-019-0178-5, PMID: 31092905

[ref35] Jeffries P. Gianinazzi S. Perotto S. Turnau K. Barea J. M. (2003). The contribution of arbuscular mycorrhizal fungi in sustainable maintenance of plant health and soil fertility. Biol. Fertil. Soils 37, 1–16. doi: 10.1007/s00374-002-0546-5

[ref36] Jing Y. Wang Y. Liu S. Zhang X. Wang Q. Liu K.. (2019). Interactive effects of soil warming, throughfall reduction, and root exclusion on soil microbial community and residues in warm-temperate oak forests. Appl. Soil Ecol. 142, 52–58. doi: 10.1016/j.apsoil.2019.05.020

[ref37] Jurburg S. D. Nunes I. Stegen J. C. Le Roux X. Priemé A. Sørensen S. J.. (2017). Autogenic succession and deterministic recovery following disturbance in soil bacterial communities. Sci. Rep. 7, 1–11. doi: 10.1038/srep45691 28383027 PMC5382530

[ref38] Kaiser M. Kleber M. Berhe A. A. (2015). How air-drying and rewetting modify soil organic matter characteristics: an assessment to improve data interpretation and inference. Soil Biol. Biochem. 80, 324–340. doi: 10.1016/j.soilbio.2014.10.018

[ref39] Kempf B. Bremer E. (1998). Uptake and synthesis of compatible solutes as microbial stress responses to high-osmolality environments. Arch. Microbiol. 170, 319–330. doi: 10.1007/s002030050649, PMID: 9818351

[ref40] Kiers E. T. Duhamel M. Beesetty Y. Mensah J. A. Franken O. Verbruggen E.. (2011). Reciprocal rewards stabilize cooperation in the mycorrhizal symbiosis. Science 333, 880–882. doi: 10.1126/science.1208473, PMID: 21836016

[ref41] Knegt B. Jansa J. Franken O. Engelmoer D. J. P. Werner G. D. A. Bücking H.. (2016). Host plant quality mediates competition between arbuscular mycorrhizal fungi. Fungal Ecol. 20, 233–240. doi: 10.1016/j.funeco.2014.09.011

[ref42] Köster K. Berninger F. Lindén A. Köster E. Pumpanen J. (2014). Recovery in fungal biomass is related to decrease in soil organic matter turnover time in a boreal fir chronosequence. Geoderma 235-236, 74–82. doi: 10.1016/j.geoderma.2014.07.001

[ref43] Li D. Liu C. M. Luo R. Sadakane K. Lam T. W. (2015). MEGAHIT: an ultra-fast single-node solution for large and complex metagenomics assembly via succinct de Bruijn graph. Bioinformatics 31, 1674–1676. doi: 10.1093/bioinformatics/btv033, PMID: 25609793

[ref44] Liu C. Jin Y. Hu Y. Tang J. Xiong Q. Xu M.. (2019). Drivers of soil bacterial community structure and diversity in tropical agroforestry systems. Agric. Ecosyst. Environ. 278, 24–34. doi: 10.1016/j.agee.2019.03.015

[ref45] Malamy J. E. (2005). Intrinsic and environmental response pathways that regulate root system architecture. Plant Cell Environ. 28, 67–77. doi: 10.1111/j.1365-3040.2005.01306.x, PMID: 16021787

[ref46] Marro N. Grilli G. Soteras F. Caccia M. Longo S. Cofré N.. (2022). The effects of arbuscular mycorrhizal fungal species and taxonomic groups on stressed and unstressed plants: a global meta-analysis. New Phytol. 235, 320–332. doi: 10.1111/nph.18102, PMID: 35302658

[ref47] Mason P. A. Wilson J. (1994). “Harnessing symbiotic associations: vesicular-arbuscular mycorrhizas,” in Tropical Trees: The Potential for Domestication and the Rebuilding of Forest Resources. eds. Leakey R. R. B. Newton A. C. (London: HMSO), 165–175.

[ref48] Menezes K. M. Silva D. K. Queiroz M. A. Félix W. P. Yano-Melo A. M. (2016). Arbuscular mycorrhizal fungal communities in buffelgrass pasture under intercropping and shading systems in Brazilian semiarid conditions. Agric. Ecosyst. Environ. 230, 55–67. doi: 10.1016/j.agee.2016.05.024

[ref49] Meng L. Zhang A. Wang F. Han X. Wang D. Li S. (2015). Arbuscular mycorrhizal fungi and rhizobium facilitate nitrogen uptake and transfer in soybean/maize intercropping system. Front. Plant Sci. 6:339. doi: 10.3389/fpls.2015.00339 26029236 PMC4429567

[ref50] Oksanen J. Blanchet F. G. Friendly M. Kindt R. Legendre P. McGlinn D.. (2019). Vegan: Community ecology package, version 2.5–6.

[ref51] Oren A. (2008). Microbial life at high salt concentrations: phylogenetic and metabolic diversity. Saline Syst. 4, 2–13. doi: 10.1186/1746-1448-4-2 18412960 PMC2329653

[ref52] Page A. Miller R. Keeney D. (1982). Methods of Soil Analysis, Part 2. Chemical and Microbiological Properties. Madison, WI, American Society of Agronomy, Inc., Soil Science Society of America.

[ref53] Peerawat M. Blaud A. Trap J. Chevallier T. Alonso P. Gay F.. (2018). Rubber plantation ageing controls soil biodiversity after land conversion from cassava. Agric. Ecosyst. Environ. 257, 92–102. doi: 10.1016/j.agee.2018.01.034

[ref54] Philippot L. Raaijmakers J. M. Lemanceau P. van Der Putten W. H. (2013). Going back to the roots: the microbial ecology of the rhizosphere. Nat. Rev. Microbiol. 11, 789–799. doi: 10.1038/nrmicro3109, PMID: 24056930

[ref55] Piton G. Foulquier A. Martinez-García L. B. Legay N. Arnoldi C. Brussaard L.. (2021). Resistance–recovery trade-off of soil microbial communities under altered rain regimes: an experimental test across European agroecosystems. J. Appl. Ecol. 58, 406–418. doi: 10.1111/1365-2664.13774

[ref56] R Core Team (2020). R: a language and environment for statistical computing.

[ref57] Radhakrishnan S. Varadharajan M. (2016). Status of microbial diversity in agroforestry systems in Tamil Nadu, India. J. Basic Microbiol. 56, 662–669. doi: 10.1002/jobm.201500639, PMID: 26924716

[ref58] Remison S. U. Snaydon R. W. (1980). A comparison of root competition and shoot competition between Dactylis glomerata and Holcus lanatus. Grass Forage Sci. 35, 183–187. doi: 10.1111/j.1365-2494.1980.tb01510.x

[ref59] Robeson M. S. King A. J. Freeman K. R. Birky C. W. Martin A. P. Schmidt S. K. (2011). Soil rotifer communities are extremely diverse globally but spatially autocorrelated locally. Proc. Natl. Acad. Sci. U. S. 108, 4406–4410. doi: 10.1073/pnas.1012678108 PMC3060258 21368117

[ref60] Rodrigues J. L. M. Pellizari V. H. Mueller R. Baek K. Da Jesus E. C. Paula F. S.. (2013). Conversion of the Amazon rainforest to agriculture results in biotic homogenization of soil bacterial communities. Proc. Natl. Acad. Sci. U. S. A. 110, 988–993. doi: 10.1073/pnas.1220608110, PMID: 23271810 PMC3549139

[ref61] Sato K. Suyama Y. Saito M. Sugawara K. (2005). A new primer for discrimination of arbuscular mycorrhizal fungi with polymerase chain reaction-denature gradient gel electrophoresis. Grassl. Sci. 51, 179–181. doi: 10.1111/j.1744-697X.2005.00023.x

[ref62] Schimel J. Balser T. C. Wallenstein M. (2007). Microbial stress-response physiology and its implications for ecosystem function. Ecology 88, 1386–1394. doi: 10.1890/06-0219, PMID: 17601131

[ref63] Sheng M. Chen X. Zhang X. Hamel C. Cui X. Chen J.. (2017). Changes in arbuscular mycorrhizal fungal attributes along a chronosequence of black locust (*Robinia pseudoacacia*) plantations can be attributed to the plantation-induced variation in soil properties. Sci. Total Environ. 599-600, 273–283. doi: 10.1016/j.scitotenv.2017.04.199 28477484

[ref64] Shukla A. Kumar A. Jha A. Chaturvedi O. P. Prasad R. Gupta A. (2009). Effects of shade on arbuscular mycorrhizal colonization and growth of crops and tree seedlings in Central India. Agrofor. Syst. 76, 95–109. doi: 10.1007/s10457-008-9182-x

[ref65] Simon L. Lalonde M. Bruns T. D. (1992). Specific amplification of 18S fungal ribosomal genes from vesicular arbuscular endomycorrhizal fungi colonizing roots. Appl. Environ. Microbiol. 58, 291–295. doi: 10.1128/aem.58.1.291-295.1992, PMID: 1339260 PMC195206

[ref66] Souza T. (2015). “Glomeromycota classification,” in Handbook of Arbuscular Mycorrhizal Fungi (Cham: Springer International Publishing), 87–128.

[ref67] Sýkorová Z. Ineichen K. Wiemken A. Redecker D. (2007). The cultivation bias: different communities of arbuscular mycorrhizal fungi detected in roots from the field, from bait plants transplanted to the field, and from a greenhouse trap experiment. Mycorrhiza 18, 1–14. doi: 10.1007/s00572-007-0147-0, PMID: 17879101

[ref68] Tamburini G. Bommarco R. Wanger T. C. Kremen C. van der Heijden M. G. Liebman M.. (2020). Agricultural diversification promotes multiple ecosystem services without compromising yield. Sci. Adv. 6:eaba1715. doi: 10.1126/sciadv.aba1715, PMID: 33148637 PMC7673676

[ref69] Torrecillas E. Alguacil M. M. Roldan A. (2012). Host preferences of arbuscular mycorrhizal fungi colonizing annual herbaceous plant species in semiarid Mediterranean prairies. Appl. Environ. Microbiol. 78, 6180–6186. doi: 10.1128/AEM.01287-12, PMID: 22752164 PMC3416610

[ref70] Tscharntke T. Clough Y. Bhagwat S. A. Buchori D. Faust H. Hertel D.. (2011). Multifunctional shade-tree management in tropical agroforestry landscapes–a review. J. Appl. Ecol. 48, 619–629. doi: 10.1111/j.1365-2664.2010.01939.x

[ref71] Turrini A. Avio L. Giovannetti M. Agnolucci M. (2018). Functional complementarity of arbuscular mycorrhizal fungi and associated microbiota: the challenge of translational research. Front. Plant Sci. 9:1407. doi: 10.3389/fpls.2018.01407, PMID: 30319670 PMC6166391

[ref72] van Dam N. M. Bouwmeester H. J. (2016). Metabolomics in the rhizosphere: tapping into belowground chemical communication. Trends Plant Sci. 21, 256–265. doi: 10.1016/j.tplants.2016.01.008, PMID: 26832948

[ref73] van Tuinen D. Tranchand E. Hirissou F. Wipf D. Courty P. E. (2020). Carbon partitioning in a walnut-maize agroforestry system through arbuscular mycorrhizal fungi. Rhizosphere 15:100230. doi: 10.1016/j.rhisph.2020.100230

[ref74] Verbruggen E. Van Der HEIJDEN M. G. Weedon J. T. Kowalchuk G. A. Röling W. F. (2012). Community assembly, species richness and nestedness of arbuscular mycorrhizal fungi in agricultural soils. Mol. Ecol. 21, 2341–2353. doi: 10.1111/j.1365-294X.2012.05534.x, PMID: 22439851

[ref75] Veresoglou S. D. Caruso T. Rillig M. C. (2013). Modelling the environmental and soil factors that shape the niches of two common arbuscular mycorrhizal fungal families. Plant Soil 368, 507–518. doi: 10.1007/s11104-012-1531-x

[ref76] Ward P. R. Dunin F. X. Micin S. F. (2002). Water use and root growth by annual and perennial pastures and subsequent crops in a phase rotation. Agric. Water Manag. 53, 83–97. doi: 10.1016/S0378-3774(01)00157-3

[ref77] Wei Z. Gu Y. Friman V. P. Kowalchuk G. A. Xu Y. Shen Q.. (2019). Initial soil microbiome composition and functioning predetermine future plant health. Sci. Adv. 5:eaaw0759. doi: 10.1126/sciadv.aaw0759, PMID: 31579818 PMC6760924

[ref78] White T. J. Bruns T. Lee S. J. W. T. Taylor J. (1990). “Amplification and direct sequencing of fungal ribosomal RNA genes for phylogenetics,” in PCR Protocols: A Guide to Methods and Applications. eds. Innis M. A. Gelfand D. H. Sninsky J. J. White T. J. (San Diego, CA: Academic Press), 315e322.

[ref79] Wipf D. Krajinski F. van Tuinen D. Recorbet G. Courty P. E. (2019). Trading on the arbuscular mycorrhiza market: from arbuscules to common mycorrhizal networks. New Phytol. 223, 1127–1142. doi: 10.1111/nph.15775, PMID: 30843207

[ref80] Yao H. Jiao X. Wu F. (2006). Effects of continuous cucumber cropping and alternative rotations under protected cultivation on soil microbial community diversity. Plant Soil 284, 195–203. doi: 10.1007/s11104-006-0023-2

[ref81] Yao Z. Xing J. Gu H. Wang H. Wu J. Xu J.. (2016). Development of microbial community structure in vegetable-growing soils from open-field to plastic-greenhouse cultivation based on the PLFA analysis. J. Soils Sediments 16, 2041–2049. doi: 10.1007/s11368-016-1397-2

[ref84] Zhang D. Zhang L. Liu J. Han S. Wang Q. Evers J.. (2014). Plant density affects light interception and yield in cotton grown as companion crop in young jujube plantations. Field Crops Res. 169, 132–139. doi: 10.1016/j.fcr.2014.09.001

[ref85] Zhang Z. Zhang J. Xu G. Zhou L. Li Y. (2019b). b. Arbuscular mycorrhizal fungi improve the growth and drought tolerance of Zenia insignis seedlings under drought stress. New For. 50, 593–604. doi: 10.1007/s11056-018-9681-1

[ref86] Zhang Y. Zheng N. Wang J. Yao H. Qiu Q. Chapman S. J. (2019a). a. High turnover rate of free phospholipids in soil confirms the classic hypothesis of PLFA methodology. Soil Biol. Biochem. 135, 323–330. doi: 10.1016/j.soilbio.2019.05.023

[ref87] Zhu Y. G. Miller R. M. (2003). Carbon cycling by arbuscular mycorrhizal fungi in soil–plant systems. Trends Plant Sci. 8, 407–409. doi: 10.1016/S1360-1385(03)00184-5, PMID: 13678905

