# Supplementary material for: Arbuscular mycorrhizal fungi contribute to wheat yield in an agroforestry system with different tree ages
Source: Front Microbiol. 2022 Nov 15;13:1024128. doi: 10.3389/fmicb.2022.1024128 (PMC9705347; doi:10.3389/fmicb.2022.1024128)
Supplement: Supplementary file 1 [file Data_Sheet_1.docx]

Arbuscular mycorrhizal fungi contribute to wheat yield in an agroforestry system with different tree ages

**Xu Qiao^1^**^†^**, Tao Sun^2,3,4^**^†^**, Lihua Xue^2^, Junjie Lei^2^, Li Xiao^2^, Heng Zhang^2,3*^, Jiyu Jia^1,5*^, Shuikuan Bei^1,5^**

^1^Institute of Medicinal Plant Development, Peking Union Medical College, Chinese Academy of Medical Sciences, Beijing 100193, China

^2^Key Laboratory of Desert-Oasis Crop Physiology，Ecology and Cultivation，MOARA/Institute of Grain Crops, Xinjiang Academy of Agricultural Sciences, Urumqi, Xinjiang 830091, China

^3^College of Water Conservancy and Civil Engineering, Xinjiang Agricultural University, Urumqi 830052, China

^4^Institute of Agricultural Resources and Regional Planning, Chinese Academy of Agricultural Sciences, 12 Zhongguancun South St., Haidian District, Beijing 100081, PR China

^5^College of Resources and Environmental Sciences; National Academy of Agriculture Green Development; Key Laboratory of Plant-Soil Interactions, Ministry of Education; China Agricultural University, Beijing 100193, China

^†^These authors contributed equally to this work.

*** Correspondence:**Jiyu Jia: jiajiyu20110909@163.com;
Heng Zhang: 471767070@qq.com


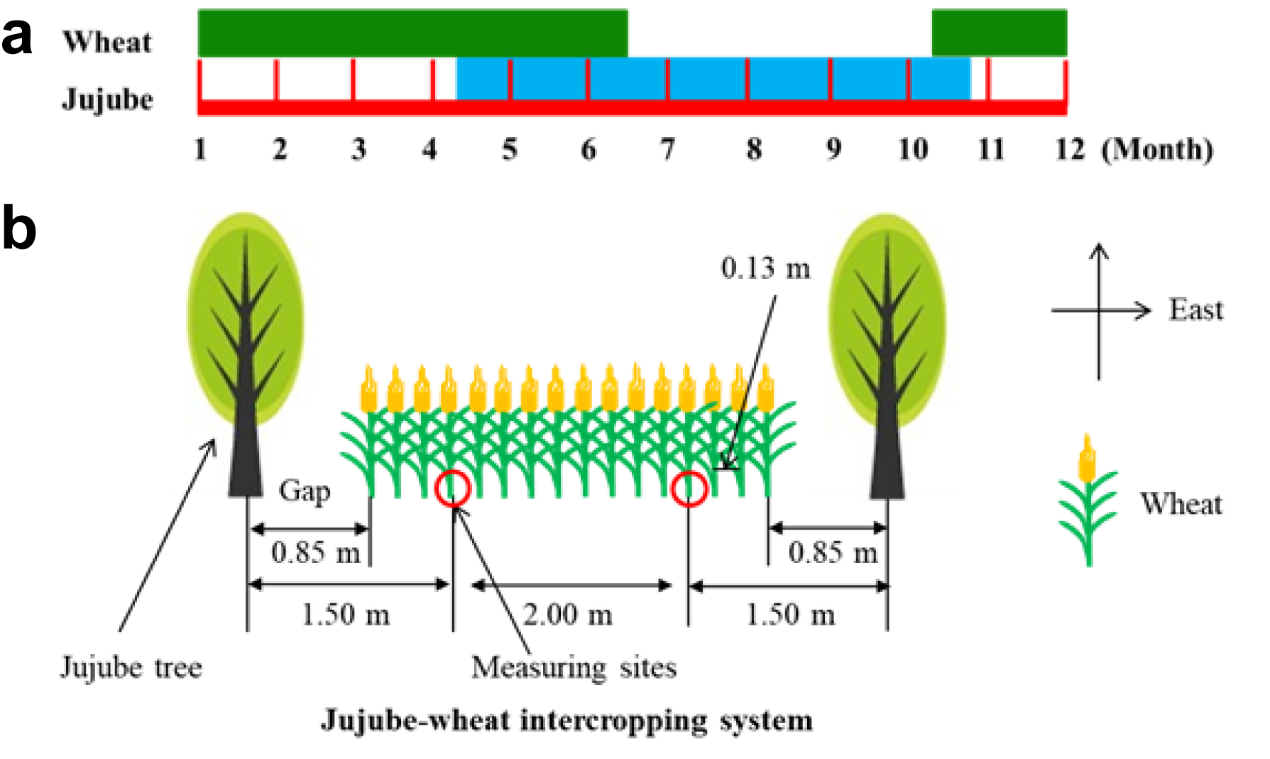


Fig.S1. Growth stage of wheat and jujube tree in the field (a). Schematic illustration of planting patterns in jujube tree-wheat based intercropping systems. Jujube trees were planted in a north–south orientation (b).


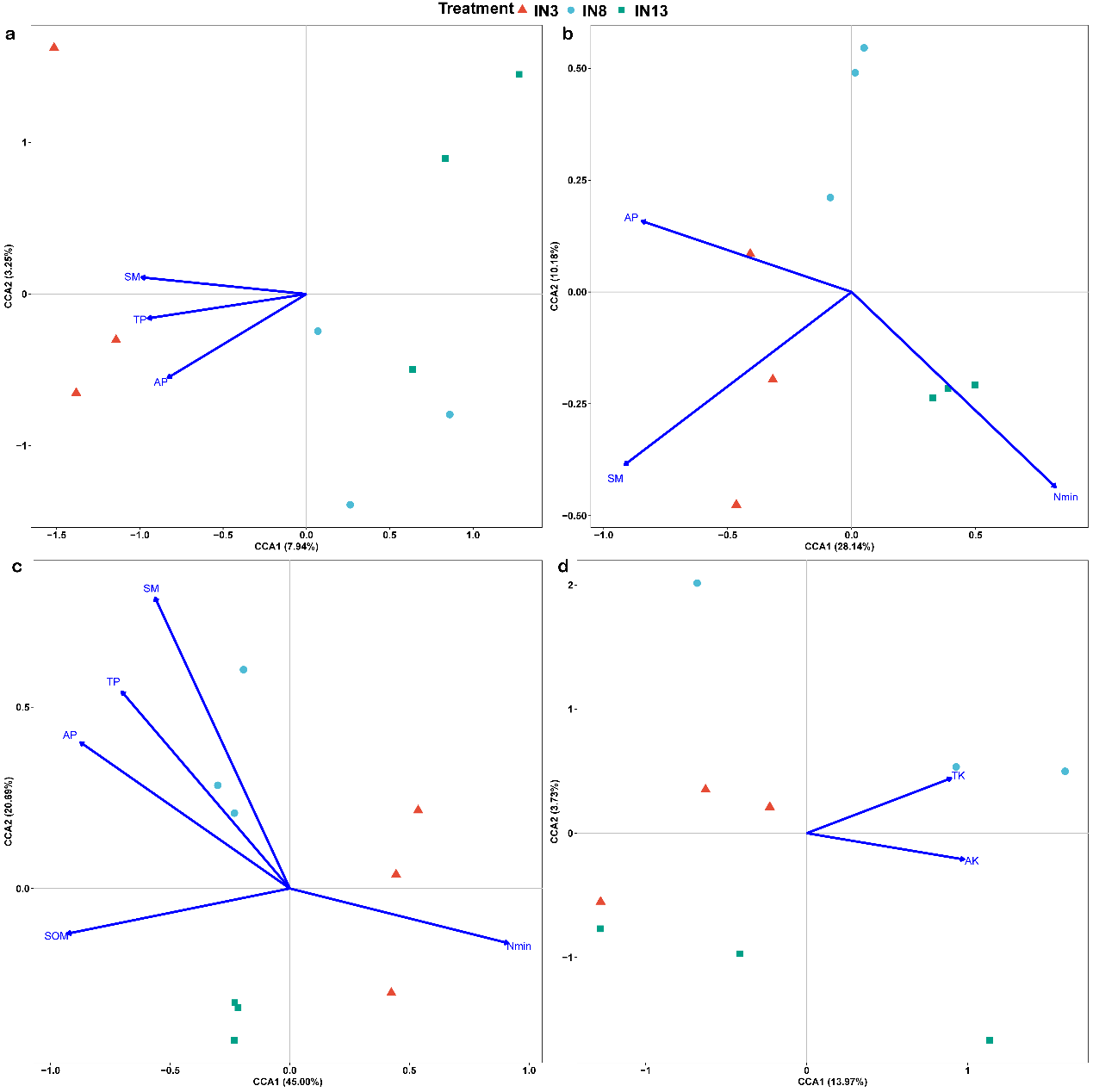


Fig.S2. Effects of soil characteristics on the distribution of soil microbial community (a) and AM fungal community of soil (b) wheat root (c) and jujube root (d) by redundancy analysis.


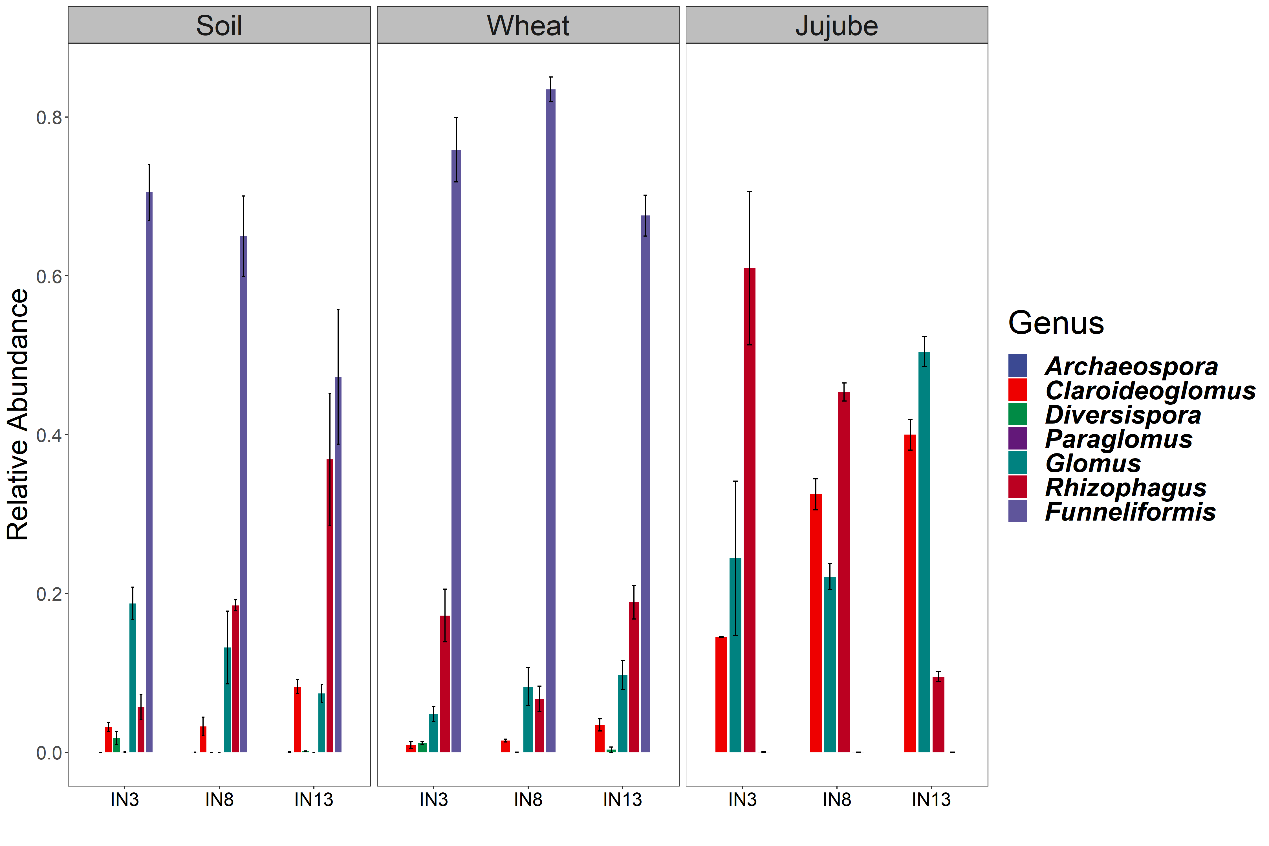


Fig.S3. relative abundance of AM fungi on genus level across different treatment in soil, wheat root and jujube root.


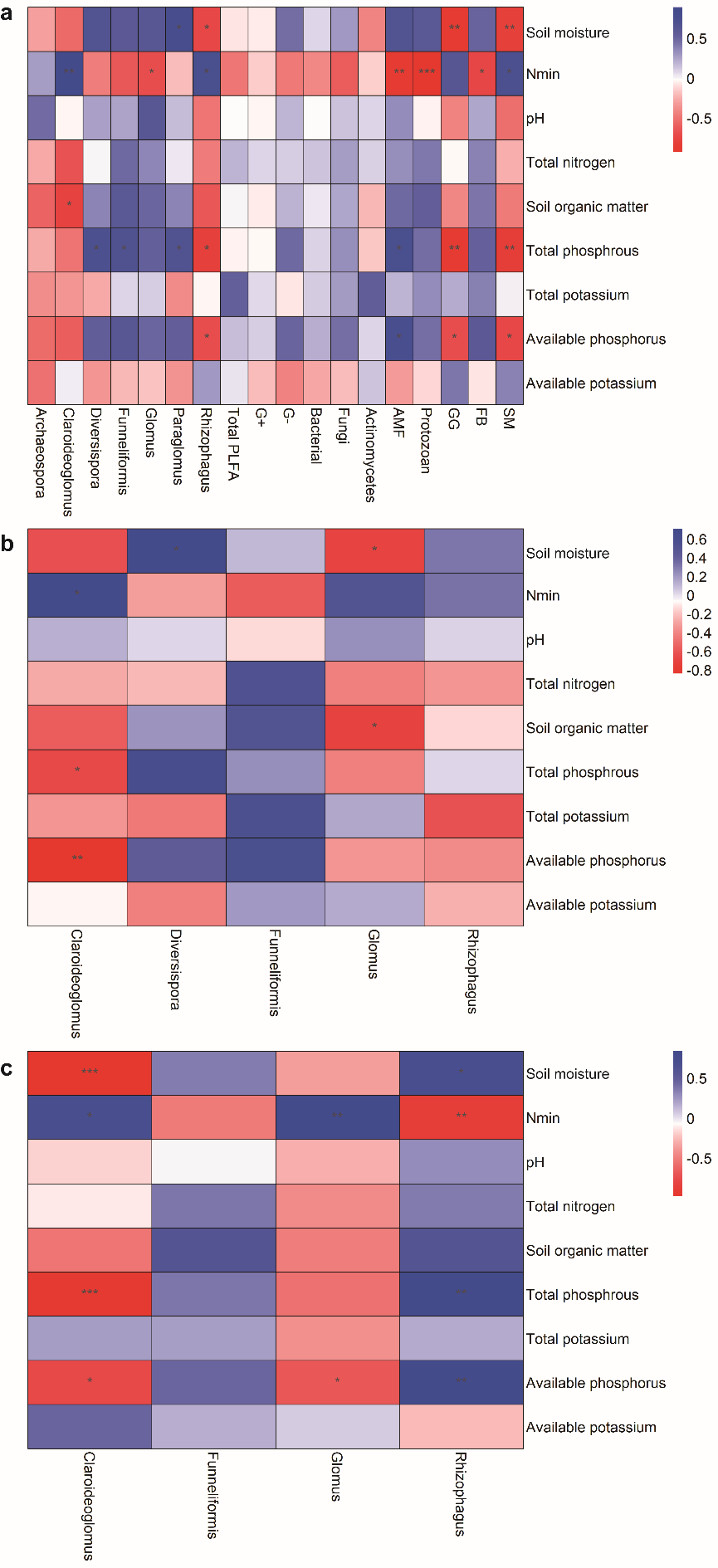


Fig. S4. Pearson correlations between soil microbiomes, relative abundance of AM Fungi on genus level and soil parameters in soil (a), wheat root (b), and jujube root (c).


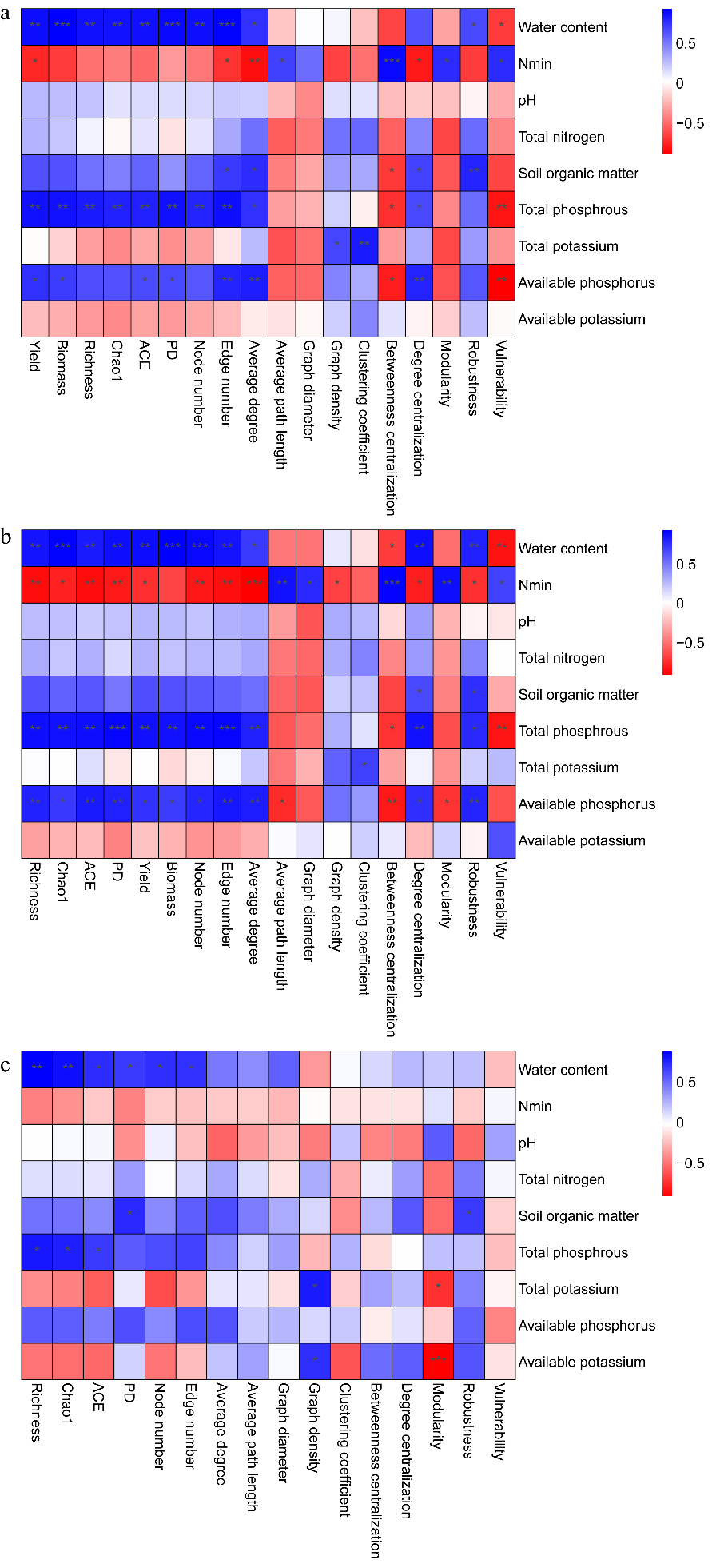


Fig. S5. Pearson correlations between soil physicochemical parameters and α diversity, topological parameters of arbuscular mycorrhizal fungi in soil (a), wheat (b), jujube (c).

Table S1. The basic information of jujube trees. DBH: diameter at breast height (cm).

| Fruiter | Spacing (m) | Age (yr) | DBH (cm) | Trunk (m) | Height (m) | Crown width (m) |
| --- | --- | --- | --- | --- | --- | --- |
| Jujube | 1.5×5.0 | 3 | 2.4 | 0.6 | 1.2 | 0.4-0.6 |
| Jujube | 1.5×5.0 | 8 | 8.3 | 0.4 | 2.5 | 2.2-2.3 |
| Jujube | 1.5×5.0 | 13 | 11.7 | 0.5 | 2.6 | 2.5-2.7 |

Table S2. Soil physicochemical parameters in different tree ages

|  | IN3 | IN8 | IN13 |
| --- | --- | --- | --- |
| Soil moisture (%) | 17.08±1.12a | 11.18±1.30ab | 9.99±1.13c |
| Inorganic nitrogen (mg/kg) | 8.08±2.81b | 8.27±0.99b | 17.04±2.21a |
| pH | 8.72±0.05a | 8.62±0.31a | 8.57±0.23a |
| Total nitrogen (g/kg) | 1.21±0.01a | 1.27±0.19a | 1.12±0.07a |
| Soil organic matter (g/kg) | 17.15±0.22a | 16.37±2.45a | 14.72±0.47b |
| Total phosphorus (g/kg) | 1.27±0.02a | 1.15±0.05b | 1.10±0.02b |
| Total potassium (g/kg) | 19.57±0.68a | 19.24±0.70b | 19.53±0.91b |
| Available phosphorus (mg/kg) | 33.40±1.21a | 31.53±0.75ab | 28.90±1.85bc |
| Available potassium (mg/kg) | 115.30±5.05ab | 127.97±15.45a | 124.10±15.12a |

Table S3. Wheat yield and aboveground biomass in different tree ages’ intercropping system

| Treat | Yield (Mg/ha) | Aboveground biomass (kg/ha) |
| --- | --- | --- |
| IN3 | 8.23±0..25a | 8364±439.80a |
| IN8 | 7.02±0.13b | 4832.33±322.21b |
| IN13 | 6.31±0.17c | 3917.00±86.64b |

Table S4. PLFA diversity in different tree ages’ intercropping system

| Treatment | Richness | Shannon | Simpson | Pielou |
| --- | --- | --- | --- | --- |
| IN3 | 44.67±0.58a | 4.34±0.08b | 0.92±0.00b | 1.14±0.02a |
| IN8 | 47.67±1.53a | 4.53±0.08a | 0.93±0.00a | 1.17±0.02a |
| IN13 | 45.00±2.65a | 4.42±0.06ab | 0.93±0.00a | 1.16±0.01a |

Table S5. Relationship between PLFA diversity and wheat yield and biomass

|  |  | Richness | Shannon | Simpson | Pielou |
| --- | --- | --- | --- | --- | --- |
| Yield | R | -0.12 | -0.54 | -0.77 | -0.66 |
|  | p | 0.75 | 0.13 | 0.02* | 0.06 |
| Aboveground biomass | R | -0.26 | -0.63 | -0.82 | -0.67 |
|  | p | 0.50 | 0.07 | 0.01** | 0.05* |

Table S6. Mycorrhizal colonization rate in wheat root and jujube root in different tree ages’ intercropping system

|  | Wheat root | | jujube root | |
| --- | --- | --- | --- | --- |
|  | M (%) | A (%) | M (%) | A (%) |
| IN3 | 27.24±0.99 | 8.06±2.73 | 5.78±2.07 | - |
| IN8 | 31.37±0.92 | 10.07±1.01 | 5.61±1.15 | - |
| IN13 | 32.00±1.07 | 9.46±1.79 | 4.22±0.77 | - |
